# Identification and Characterization of ATP-Binding Cassette Transporters in *Chlamydomonas reinhardtii*

**DOI:** 10.3390/md20100603

**Published:** 2022-09-25

**Authors:** Xiangyu Li, Xiaolian Li, Xingcai Yang, Chengxiang Lan, Ying Huang, Bin Jia

**Affiliations:** 1 Guangdong Technology Research Center for Marine Algal Bioengineering, Guangdong Provincial Key Laboratory for Plant Epigenetics, Shenzhen Engineering Laboratory for Marine Algal Biotechnology, Longhua Innovation Institute for Biotechnology, College of Life Sciences and Oceanography, Shenzhen University, Shenzhen 518060, China; 2 College of Physics and Optoelectronic Engineering, Shenzhen University, Shenzhen 518060, China

**Keywords:** microalgae, ABC transporters, heavy metal transport, lipid transport

## Abstract

Microalgae are promising microorganisms used to produce value-added products or to develop sustainable approaches for environmental remediation. The ATP-binding cassette proteins (ABCs) of *Chlamydomonas reinhardtii* have been characterized as indispensable transporters for CO_2_ concentrating mechanism, lipid biosynthesis, and heavy metal sequestration. However, few microalgal ABC proteins have been studied compared with higher plants or non-photosynthetic microorganisms. This study performed a genome-wide, evolutionary, and transcriptomic survey of *C. reinhardtii* ABC proteins (CrABCs). A total of 75 *CrABCs* were identified and classed into eight ABC subfamilies, from ABCA to ABCI. We found that no whole or partial genome duplication events occurred in *C. reinhardtii* after the ancient endosymbiosis events, but gene duplications occurred in a small range of chromosomal regions, which forced ABC family expansion. Abundant light, abscisic acid, and jasmonic acid response *cis*-elements were mapped in the *CrABC* promoters, coinciding with the evolutionary history of hormone signaling in *Chlorophyta*. The expression survey under light/dark rhythms revealed a close bond of *CrABCs* with cell division and development. A broad study of CrABCs supported their expected roles in heavy metal detoxification, lipid metabolism, and environmental adaptation. Moreover, the evolutionary and expression survey predicted the functions of unknown CrABCs, which are elaborated in the text. Two half-size CrABCGs—CrABCG3 and CrABCG26—were described as plasma-membrane transporters that might participate in lipidic compound secretion. This study provides fundamental and exhaustive information about *CrABCs*, which are indispensable for the functional elucidation of ABC proteins in microalgae.

## 1. Introduction

**Figure 1:**
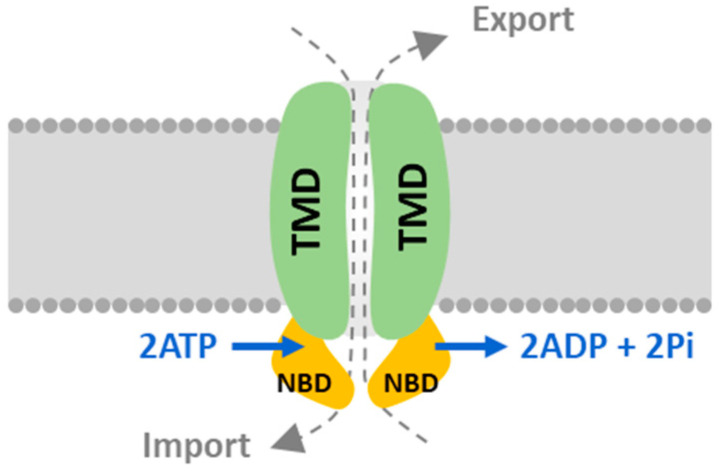
The schematic of the ABC transporter. A functional ABC transporter is constituted by two sets of TMDs and NBDs and functions as either an importer or an exporter. TMD (green) is the hydrophobic transmembrane domain. NBD (orange) is for ATP binding and hydrolysis. The NBDs hydrolyze ATP and drive conformational changes in TMDs; thus, the substrates cross the membrane.

ATP-binding cassette transporters (ABCs) constitute one of the ancient families in the biological kingdom and translocate molecules driven by ATP hydrolysis. As controllable channels embedded in lipid membranes, they may act as either importers and/or exporters for a wide range of molecules, such as xenobiotics, hormones, sugars, amino acids, and ions [1,2]. ABC transporters (ABCs) need two conserved domains, i.e., the nucleotide-binding domain (NBD) and the transmembrane domain (TMD) (Figure 1). The NBD is a conserved domain constituted by the Walker A, Q-loop, Walker B, D-loop, and H-loop motifs essential for ATP binding and hydrolysis [3,4]. TMD is less conserved, with several α-helices that form various ligand binding sites for specific substrates during evolution and that determine the transport affinity of multiple chemicals. 

There are eight subfamilies formed in the plant ABC family with different distributions of NBDs and TMDs, namely, ABCA, ABCB, ABCC, ABCD, ABCE, ABCF, ABCG, and ABCI [5]. Three typical structures are found in the ABC protein family. Full transporters have two NBDs and two TMDs. Half transporters, having one TMD and one NBD, dimerize as heterodimers or homodimers similar to full transporters. The last type only has an NBD (no TMD). From the ABCA to ABCD subfamilies, a forward TMD–NBD structure is generally found. Nevertheless, the ABCG subfamily encodes proteins with reverse NBD–TMD organization. ABCE and ABCF proteins have two NBDs with no TMD, and ABCI proteins have only a single NBD or accessory domains.

Microalgae are single-cell photosynthetic microorganisms naturally occurring in various habitats. As essential primary producers, microalgae are advocated to be suitable and competitive candidates for producing various biomolecules used for food, feed, and fuel applications, with the concurrent benefit of greenhouse gas sequestration [6]. Accordingly, the biotechnological applications of microalgae are exploited in three main areas: bioenergy, environmental remediation, and high-value-added products [7]. ABCs have been characterized as key contributors to these excellent traits. In *Chlamydomonas reinhardtii* (*C. reinhardtii*), CrHLA3 (Cre02.g097800) is a critical inorganic carbon (Ci) transporter that works in the CO_2_ concentrating mechanism (CCM) [8,9]. CrCds1 (Cre12g561550) and CrMRP2 (Cre17g734612) confer cadmium sequestration abilities on *C. reinhardtii* [10,11]. *CrABCA2* (Cre14g613950) facilitates intracellular lipid accumulation [12]. Even though few ABC transporters have been characterized in microalgae, they show great application potential in microalgal biotechnology. For example, ABC engineering successfully improved lipid production in microalgae [13,14,15]. 

In this study, we performed a genome-wide identification and characterization of ABCs in *C. reinhardtii*. The physical and chemical properties, chromosome location, gene structure, family duplications, phylogenetic evolution, cis-regulatory element prediction, and expression analysis of *CrABCs* were studied to provide molecular clues that further reveal the function of ABC proteins in microalgae. Our results will facilitate future biotechnological research and transporter engineering to improve performance in microalgae.

## 2. Results and Discussion

### 2.1. Identification, Classification, and Nomenclature of ATP-Binding Cassette Proteins in C. reinhardtii

A previous study carried out a genome-wide identification of *CrABCs* and found 69 putative *CrABCs* in Genome v3.0 (JGI Project ID: 16938) [16]. However, this information needs to be further updated due to incomplete annotations of the reference-grade Genome v3.0. The current *C. reinhardtii* genome has been updated to v5.6 (JGI Project ID: 1084054). Recently, a comprehensive database of algal multi-omics named PhycoCosm was developed by the US Department of Energy Joint Genome Institute [17]. There are 45 loci of ABC proteins annotated in PhycoCosm. Through artificial verification, we found a misannotated ABC gene (Cre10.g427050) in PhycoCosm. Then, we performed a genome-wide identification of *CrABCs* afresh, as described in the Materials and Methods section. Finally, we identified 73 putative ABC protein loci, as listed in Table S1, which encompassed 44 candidates of PhycoCosm and 29 novel candidates in this study.

The 75 *CrABC* loci were unevenly distributed in all of the chromosomes (Figure S1). Chromosome 17 had the largest number of *CrABC* loci, ten, followed by Chromosome 2 and 16. The shortest chromosome, Chromosome 15, harbored only a single *CrABC*. These loci encoded at least 87 *CrABC* transcripts and yielded proteins with molecular weights ranging from 31.07 kDa to 372.21 kDa (Table S1). The gene structures are shown in Figure S2. On average, each gene contained 22 exons, but the number of exons of *CrABCs* varied immensely (Figure S2). The predicted sub-localization and signal peptides of CrABCs are also summarized in Table S1.

**Figure 2:**
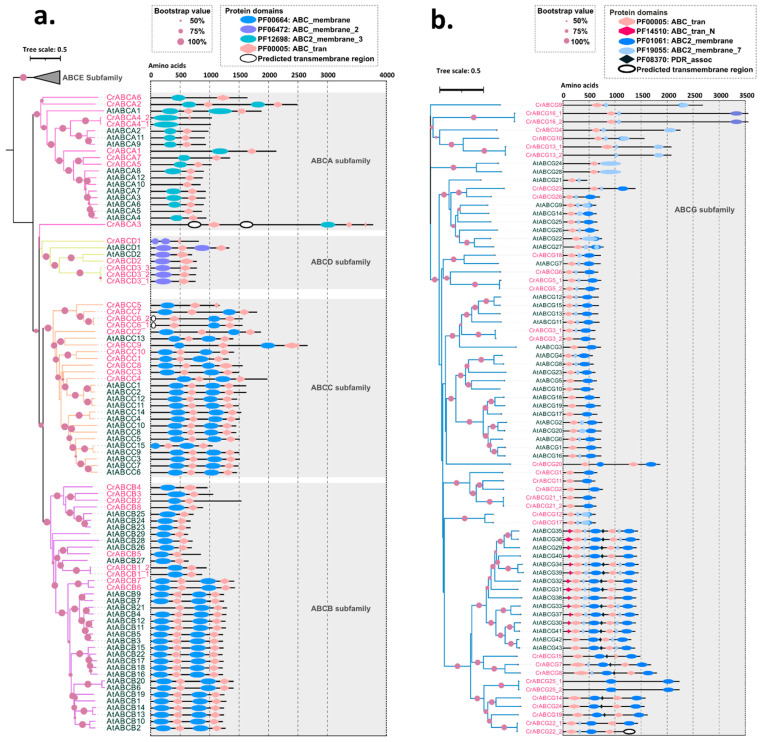
Evolutionary comparison of ABC topological structure in *C. reinhardtii* and *Arabidopsis*. (**a**) Phylogenetic tree of ABCs with TMD–NBD structures. (**b**) Phylogenetic tree of ABCs with NBD–TMD structures. The neighbor-joining tree was generated using the full-length ABC proteins with the bootstrap test of 1000 replicates, and bootstrap values > 50% are shown with purple dots of different sizes on the branches. Branches of subfamilies are marked by a different color. The leaves of *C. reinhardtii* and *Arabidopsis* are labeled with pink and black separately. The Pfam ID of NBD is PF00005. The Pfam IDs of TMD are PF00664, PF06472, PF12698, PF01061, and PF19055. ABC_trans_N (PF14510) is a conservative domain found at the N-terminus of ABC-transporter proteins. PDR_assoc (PF08370) is a conservative domain of pleiotropic drug resistance (PDR) ABC transporters.

Phylogenetic analysis of CrABC proteins was performed together with 128 *Arabidopsis* ABCs (AtABCs) (Table S2) [2]. Considering the distinct distribution of domains in ABC proteins, three phylogenetic trees were constructed separately: a phylogenic tree consisting of ABCs with TMD–NBD structures (Figure 2a), a phylogenic tree consisting of ABCs with NBD–TMD structures (Figure 2b), and a phylogenic tree consisting of ABCs with only NBDs (no TMDs) (Figure S3). Finally, 75 CrABCs were grouped into eight subfamilies (Figure 2 and Figure S3), namely 7 ABCAs, 8 ABCBs, 9 ABCCs, 3 ABCDs, 1 ABCE, 11 ABCFs, 25 ABCGs, and 9 ABCIs (Table 1). Thereafter, we performed a prediction of the transmembrane regions of ABC proteins in each subfamily using TMHMM 2.0. Generally, ABC proteins of the ABCA, ABCB, ABCC, ABCD, and ABCG subfamilies were transmembrane transporters with obvious hydrophobic helices (Supplementary file 1). The ABCE, ABCF, and ABCI subfamily proteins were soluble proteins with no transmembrane regions (Supplementary file 1). To facilitate more ABC studies, we normatively named these *CrABC* genes based on the evolutionary relationship and the consolidated nomenclature proposed by Verrier et al. [5] (Table S1). Accordingly, we numbered the *CrABCs* based on the order of their positions on the chromosomes (Figure S1).

### 2.2. Duplications of ATP-Binding Cassette Genes in C. reinhardtii

**Figure 3:**
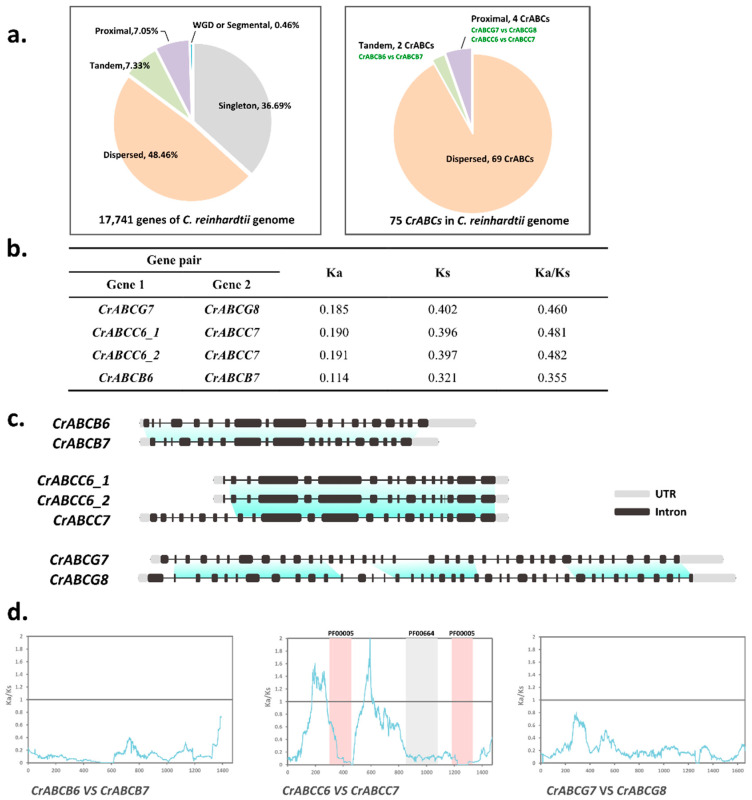
The duplications events of *CrABCs*. (**a**) Pie chart showing *C. reinhardtii* genes with different duplication types. (**b**) Ka/Ks ratio of orthologous pairs. (**c**) Gene structures of orthologous pairs. (**d**) Sliding window analysis of orthologous pairs.

It is well known that the evolutionary history of the ABC family is punctuated by gene duplication events driving the morphology diversification of plants [20,21]. Through the analysis of the duplication types of whole *C. reinhardtii* genes using MCScanX, we found that only 82 of the 17,741 genes (0.46%) were produced by segmental or whole genome duplication (Figure 3a). Whole or partial genome duplication events did not occur in *C. reinhardtii*, as shown in the genome dot plot analysis in Figure S4. Indeed, 69 of 75 *CrABCs* were dispersed duplications (Figure 3a). Only three gene pairs, namely CrABCC6 and *CrABCC7*, *CrABCB6* and *CrABCB7*, and *CrABCG7* and *CrABCG8*, arose from small-scale duplication, tandem duplication or insertion (Figure 3a). This was attested by the adjacent physical distances in the chromosomes (Figure S1 and Figure S4) and the coupled evolutionary distances in the phylogenetic tree (Figure 2a,b).

Accordingly, Ka/Ks ratios were calculated to investigate the evolutionary pressure on these duplicating pairs. All of these paralogous pairs largely encountered a purifying selection (Ka/Ks = 0.35~0.48) (Figure 3b). The highly conservative gene structure of these pairs verified that these paralogous genes resulted from recent duplication on a small scale (Figure 3c). The higher *Ks* value of *CrABCG7/8* indicates that the duplication event of the ancestral CrABCG7/8 was earlier than the duplication of *CrABCB6/7* or *CrABCC6/7* (Figure 3b). This suggests that *CrABCG7/8* encountered longer evolutionary history, which led to more various gene structures of *CrABCG7/8* compared with *CrABCB6/7* and *CrABCC6/7* (Figure 3c). Whereafter, a sliding window analysis was employed to investigate the evolutionary pressure on the coding sites or regions of paralogous genes. The whole coding regions of *CrABCB6/7* and *CrABCG7/8* underlay the intense purifying selection (Figure 3d), indicating possible functional redundancies in CrABCB6_CrABCB7 and CrABCG7_CrABCG8. The regions beyond the TMDs (PF00664) and NBDs (PF00005) of CrABCC6_CrABCC7 underwent positive selection, including the coding regions of the 181–274 amino acid residues and the 554–602 amino acid residues (Figure 3d). CrABCC6 lost the 5′ region coding for the first TMD (Supplementary file 1), which indicates a potential functional divergence between CrABCC6 and CrABCC7.

### 2.3. Comparison of ABC Structure and Function Diversity by Phylogenetic Analysis 

#### 2.3.1. ABCA Subfamily

Seven *CrABCAs* were identified in the *C. reinhardtii* genome, coding five half-size transporters and two full-size transporters. CrABCA2 and CrABCA3 were the full-size transporters. However, CrABCA3 was shown in an independent branch with low bootstrap confidence (Figure 2a). The secondary structure of CrABCA3 showed a unique TMD–NBD–TMD–TMD–NBD structure, and the C-terminal NBD of CrABCA3 was incomplete (Figure 2a). For plants, the orthologous protein of CrABCA2 commonly exists in dicotyledons, such as *Arabidopsis* [5], tomato [18], and strawberry [22], while it is lost in dicotyledons [19,23]. The existence of CrABCA2 suggests that the full-size ABCA originated from ancient microalgae but was lost in some land lineage during plant diversification. Although the conservative full-size ABCA is found in land plants, its function is unknown for now. 

Other than plants, most animal ABCAs are full-size transporters and are involved in the transport of physiologic lipid compounds [24,25]. Nevertheless, the half-size ABCAs are also proven to participate in lipid transport in plants [3,26,27], such as the ER-located AtABCA9, which is a supplier of fatty acid substrates for TAG biosynthesis during the seed-filling stage [28]. For *C. reinhardtii*, two branches of half-size ABCAs were constructed with high confidence support. One consisted of CrABCA4, AtABCA9, AtABCA2, and AtABCA11, and the other consisted of CrABCA1, CrABCA5, CrABCA7, and the rest of the half-size AtABCAs (Figure 2a). CrABCA4 (old name: CrABCA2), a homologous protein of AtABCA9, improves the TAG accumulation in *C. reinhardtii* [12,15]. Recently, AtABCA10 in another branch has also been reported as an ER-located transporter that induces TAG overaccumulation in seeds [29]. Therefore, it is tempting to speculate that more ABCAs might be involved in microalgal lipid metabolism.

#### 2.3.2. ABCB Subfamily 

Six loci of *C. reinhardtii* encode half-size ABCBs. Orthologous lineages of half-size CrABCBs were found in *Arabidopsis* (Figure 2a), suggesting that no gene expansion or loss occurred in half-size ABCBs. The phylogenetic relationships of the half-size members of CrABCBs and AtABCBs were isogenous in high supporting branches, including the branch consisting of CrABCB2, CrABCB3, CrABCB4, CrABCB8, AtABCB24, AtABCB23, and AtABCB25; the branch consisting of CrABCB5 and AtABCB26; and the branch consisting of CrABCB1 and AtABCB27 (Figure 2a). Half-size ABCBs are pivotal transporters for heavy metal resistance. CrABCB3 (old name: CrCds1) is a mitochondria-located protein and plays a pivotal role in cadmium tolerance [10]. Its orthologous lineages of *Arabidopsis*, AtABCB24, AtABCB23, and AtABCB25, are also mitochondria-localized transporters [30,31]. In particular, AtABCB25 is a vital transporter involved in the maturation of the prosthetic groups of the Fe–sulfur and molybdenum cofactors [32,33] and ensures heavy metal resistance [34]. The cadmium tolerance conferred by CrABCB3 is speculated to contribute to the intracellular Fe^2+^ homeostasis of microalgae [10], but whether CrABCB3 is essential for Fe–sulfur cofactor formation is unknown. AtABCB27, the homologous protein of CrABCB1, is a vacuolar transporter relieving *Arabidopsis* from aluminum toxicity [35]. All of this indicates that the underlying relationship of half-size ABCBs responds to heavy metal stress of microalgae.

Two full-size *ABCB* loci in *C. reinhardtii* are distinct from the expansion of full-size *ABCB* members in higher plants. Previous studies reported 21, 18, 19, 24, and 19 full-size *ABCBs* in *Arabidopsis* [5], tomato [18], strawberry [22], rice [23], and barley [19], respectively. Full-size ABCBs have attracted attention in higher plants due to their widespread function of auxin transport [36]. All of the characterized full-size AtABCBs localized to the plasma membrane for auxin transport, including AtABCB1, AtABCB4, AtABCB14, AtABCB15, AtABCB19, and AtABCB21 [37,38,39,40]. Their homologous proteins in rice show the same auxin-transport activity [41]. The family expansion of full-size *ABCBs* might drive the morphology diversification of plants. Moreover, full-size AtABCB14 is a malate importer of guard cells in stomatal regulation [42], which indicates the pleiotropic transport activity of full-size ABCBs.

#### 2.3.3. ABCC Subfamily

Unlike *Arabidopsis*, which only has full-size AtABCCs [43], we found seven full-size transporters, a half-size transporter, and a unique transporter with an NBD–TMD–NBD structure in *C. reinhardtii* (Figure 2a). Two independent branches were found in the ABCC subfamily. One branch included three CrABCCs, namely, the half-size CrABCC5, the full-size CrABCC7, and its paralogous CrABCC6. The other consisted of the rest of the full-size CrABCCs and AtABCCs (Figure 2a). Evidence shows that the functions and locations of CrABCCs are diverse in microalgae. CrABCC1 (old name: CrHLA3) is a plasma-membrane-binding transporter that takes charge of HCO_3_^−^ influx in the microalgal CCM [44,45]. CrABCC10 (old name: CrMRP2), sharing 44.74% similarity with CrABCC1, is predicted to be a vacuolar transporter conferring cadmium tolerance of microalgae [11]. ABCCs play important roles in cell detoxification in plants. The vacuolar-localized ABCCs capture xenobiotics into isolated vacuoles, including glutathione conjugates, chlorophyll catabolites, and heavy metals [46,47,48,49,50]. These homologous proteins were verified with the same ability in poplar, *Vicia sativa*, and strawberry [21,51,52]. The plasma membrane-localized transporters enhance xenobiotics efflux extracellularly, such as glyphosate efflux [53,54]. Moreover, ABCCs transport various compounds into vacuoles, such as phytohormone derivatives, flavonoids, and carotenoids [55,56,57]. Therefore, we speculated that microalgal ABCCs are mainly involved in cell detoxification except for essential roles in the CCM.

#### 2.3.4. ABCD Subfamily

Three genes were found to be *ABCDs* in *C. reinhardtii*, consistent with a few *ABCDs* found in higher plants, for example, two in *Arabidopsis*, two in tomato, one in strawberry, three in rice, and four in barley [5,18,22]. In contrast to at least one full-size transporter in higher plants [5,18,22], all ABCDs of *C. reinhardtii* were half-size transporters (Figure 2a) consistent with humans and yeast [43,44]. ABCDs are also known as peroxisomal membrane proteins (PMPs) in *Arabidopsis*, humans, and yeast. Full-size AtABCD1 supplies substrates for β-oxidation in peroxisomes [45,46,47,48,49]. Full-size *ABCD* mutations of yeast display reduced β-oxidation and cannot utilize oleate as the sole carbon source [50]. This suggests the potential function of CrABCDs in the lipid metabolism of peroxisomes.

#### 2.3.5. ABCG Subfamily

The ABCG subfamily is the largest subfamily. Twenty-six loci were found to code eight full-size transporters and eighteen half-size transporters. In particular, CrABCG25 was a unique full-size transporter with two independent TMDs but no NBD (Figure 2b). The pleiotropic drug resistance (PDR) associated domain (PF08370) was also annotated to most full-size CrABCGs. Pleiotropic drug resistance ABCGs are only identified in fungi and plants [51]. Plasma membrane-localized ABCGs show transport activity for diverse substrates, including lipid (precursors of wax, cutin, and suberin) for the formation of cell barriers [52], phytohormones for the regulation of plant development and defense [2,52], and secondary metabolites for resistance against pathogens [53,54,55]. PDR-associated ABCGs participate in cell detoxification, phytohormone transport, metabolite excretion, and biological and abiotic stress in higher plants [2,56]. No microalgal ABCGs have been characterized thus far, and the pleiotropic roles of ABCGs deserve more attention in future microalgal studies. 

#### 2.3.6. ABCE and ABCF Subfamily

There are a sole *ABCE* locus and eleven *ABCF* loci in *C. reinhardtii*. The only copy of *ABCE* is highly conserved in most eukaryotes and archaea [57,58]. Likewise, the sole ABCE found in *C. reinhardtii* has a highly conserved structure in relation to the *Arabidopsis* ABCEs (72.5% and 79.5% similarity to AtABCE1 and AtABCE2). Nevertheless, more abundant ABCF members were found in *C. reinhardtii* compared with the five ABCFs in *Arabidopsis* (Table 1). Endosymbiosis events introduce more ABCF members in algae and plants than in other eukaryotes [59], which indicates the loss of *ABCFs* as the evolution from unicellular photosynthetic organisms to multicellular plants. CrABCEs and CrABCFs harbored two conserved NBDs with no TMDs (Figure S3). ABCE is also named RNase L inhibitor [58] and possesses a conservative domain interacting with nucleic acids (PF00037 and PF04068) at the N-terminus. The loss of function of *ABCE* genes leads to lethal phenotypes in all studied species [58]. Yeast ABCE, as part of the translational apparatus, plays a role in ribosome biogenesis and reactivation for translation regulation [58]. *AtABCE1* and *AtABCE2* are involved in RNA interference (RNAi) regulation [60,61,62]. Furthermore, the plant ABCFs also participate in translation regulation [63] and DNA reparation [64]. 

#### 2.3.7. ABCI Subfamily

Nine loci recode ABCI proteins in *C. reinhardtii*. Orthologous proteins of all CrABCIs were found in *Arabidopsis* according to the phylogenetic tree (Figure S3), which indicates the ancient origins and conservative evolution of *ABCIs* from *Chlorophyta* to higher plants. AtABCI1 and AtABCI2 are components of the cytochrome c maturation complex [65]. AtABCI6, AtABCI7, and AtABCI8 constitute the Fe–sulfur cluster biogenesis complex [66]. AtABCI13, AtABCI14, and AtABCI15 interact with TGD4 and TGD5 to form a transporter complex for lipid trafficking from the endoplasmic reticulum and the chloroplast [67]. AtABCI16 and AtABCI17 play a role in aluminum tolerance [68,69,70]. AtABCI19, AtABCI20, and AtABCI21 modulate cytokinin-driven growth inhibition in young seedlings [71]. Therefore, ABCI proteins are a class of independent units forming multidomain transporters.

### 2.4. Analysis of Cis-Acting Elements in CrABC Promoters

**Figure 4:**
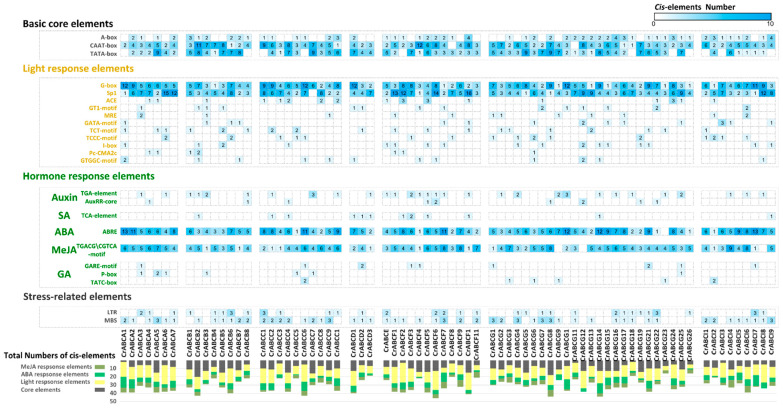
The numbers of putative *cis*-acting elements in the promoters of *CrABCs*.

*Cis*-elements that exist in promoters can affect the expression of functional genes. The 2kb upstream regions of *CrABCs* were analyzed for *cis*-elements using the PlantCARE database. As a result, it was found that *CrABC* promoters contained basic core elements (TATA-box and CAAT-box), light response elements (G-box and Sp1), ABA response elements (ABRE), and MejA response elements (TGACG-motif) universally (Figure 4). However, the positions and the numbers of *cis*-elements varied, suggesting the important roles of light and hormones in the regulation of *CrABCs*. Since the light rhythm is the basic force driving the cell and metabolic differentiation of photosynthetic microalgae, these light response elements may be essential for the daily expression of *CrABCs*. Additionally, it is not surprising that ABA and MeJA response element was the most abundant elements in the *CrABC* promoters, since ABA and JA signaling pathways arose in microalgae while auxin, cytokinin, and gibberellin signaling emerged after the *Charophyte* lineages [72]. These essential motivations, including light and ABA, are important regulators of microalgae growth and development, and these *CrABCs* would be some of the underlying responders in this process.

### 2.5. Expression Survey of CrABCs in C. reinhardtii 

#### 2.5.1. Cell Growth and Development under Daily Rhythms

**Figure 5:**
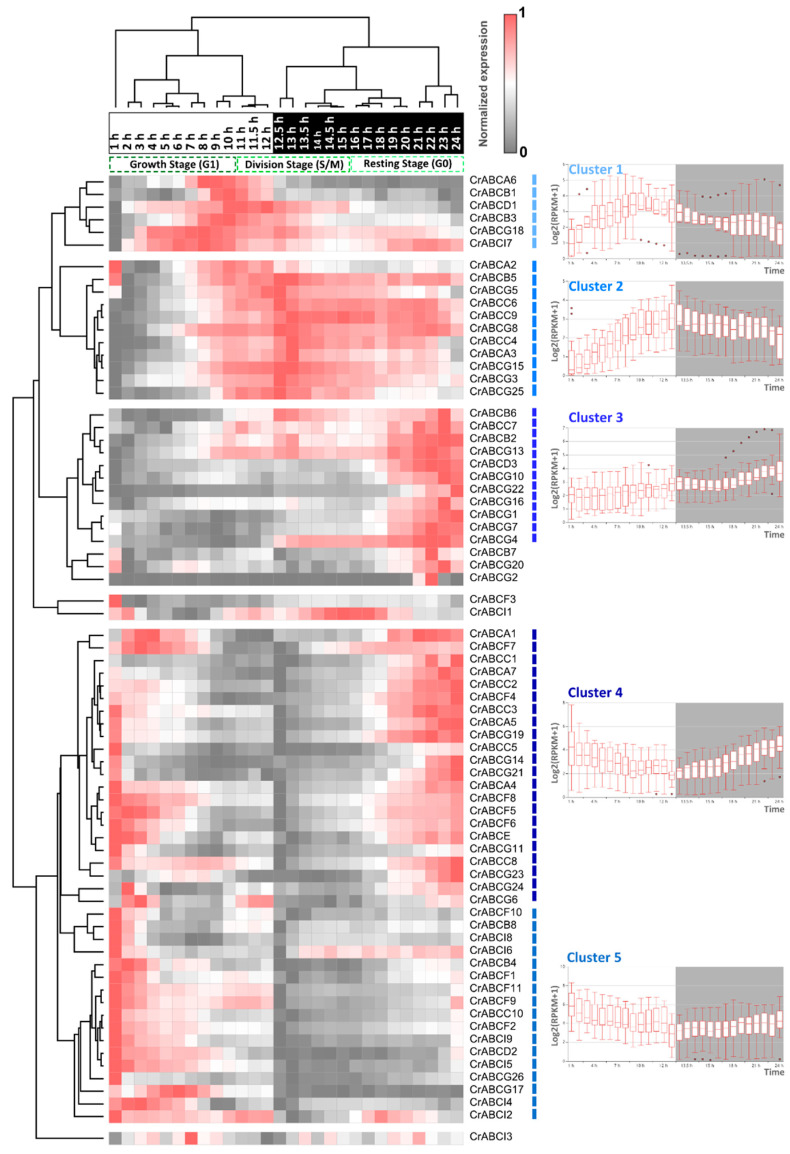
Dynamic expression of *CrABCs* during cell proliferation and development. The expression data were retrieved from Zones et al. (2015) [73]. Relative times and cell cycle stages are labeled above the heatmap. The white and black background of relative times indicate the light and dark periods, respectively. The box plots show the expressing trends of *CrABCs* in each cluster under light (white background) and dark (grey background) rhythms.

We used the bulk transcriptomes under a 12-h-light/12-h-dark daily cycle from highly synchronized populations of *C. reinhardtii* to investigate the expression pattern of *CrABCs* underlying cell development and division under a diurnal rhythm [73]. The expression level of *CrABC* genes is presented in a heatmap (Figure 5). A total of 71 out of 73 *CrABCs* were detected in time-course transcriptomes representing the cell-development courses (Figure 5 and Table S3). The expression pattern of *CrABCs* shifted along time courses representing the cell proliferation and development processes of *C. reinhardtii*. The expression pattern of these *CrABCs* was clustered into five main groups by the Hierarchical clustering algorithm. We found that the expression of Cluster 1 was higher from the later period of the growth stage (G1) to the early period of the division stage (S/M). Cluster 2 was highly expressed during S/M and the resting stage (G0). A more robust expression of Cluster 3 was detected in the later period of G0. Additionally, Cluster 4 was mainly expressed from the later period of G0 to the early period of G1. Cluster 5 was up-regulated in the early period of G1. This suggests that the abundance of *CrABC* transcripts highly correlates with the cell cycle. For example, the expression peaks of peroxisomal *CrABCD1* (Cluster 1), *CrABCD3* (Cluster 3), and *CrABCD2* (Cluster 5) were staggered. *CrABCD3* with the highest transcriptional level was persistently up-regulated in the G0 stage of the dark phase, in contrast to the changeless expression at G1 under light (Table S3). The storage TAG is remobilized at night for cell development [74,75], indicating that CrABCD3 is a pivotal long-chain acyl-CoA transporter for lipid catabolism. Additionally, the CCM-related *CrABCC1* (Cluster 4) showed a higher expression at the 21–24 h stage of the dark phase than in the light phase, which might be the preparation for the photosynthesis of G0. The more energetic expression of *CrABCC1* at night indicates that the internal clock also supervises the expression of the Ci transporter, in addition to external stimulation (e.g., high light or low CO_2_), as reported previously [76]. Therefore, the oscillatory expression of transporters may be adaptive to the physiological need for cell development under daily rhythms.

#### 2.5.2. High-Concentration CO_2_ Conditions 

Since the CCM of microalgal photosynthesis needs the help of plasma-membrane CrABCC1, we investigated the influence of the CO_2_ concentration on the expression of *CrABCs* (Table S4). *CrABCC1* was remarkably repressed by high-concentration CO_2_ cultivation, regardless of whether this was under ST or LT conditions, which is consistent with a previous study [76]. Additionally, LT conditions even led to the silencing of *CrABCC1* (FPKM = ~0.30 in LT groups compared with FPKM > ~150 in control groups). Besides *CrABCC1*, no other ABC genes were repressed in ST groups, but three transporters—*CrABCG2*, *CrABCG6* and *CrABCG7*—were markedly induced. Under LT conditions, *CrABCG6* and *CrABCG7* were observably up-regulated as well. At the same time, the other eight and ten *CrABCs* were up- and down-regulated respectively in the LT groups. Long-term acclimatization changed the expression pattern of more *CrABCs* compared with short-term treatment, which indicates the crucial roles of *CrABCs* in the long-term acclimatization of microalgae. 

#### 2.5.3. Lead and Cadmium Stress

**Figure 6:**
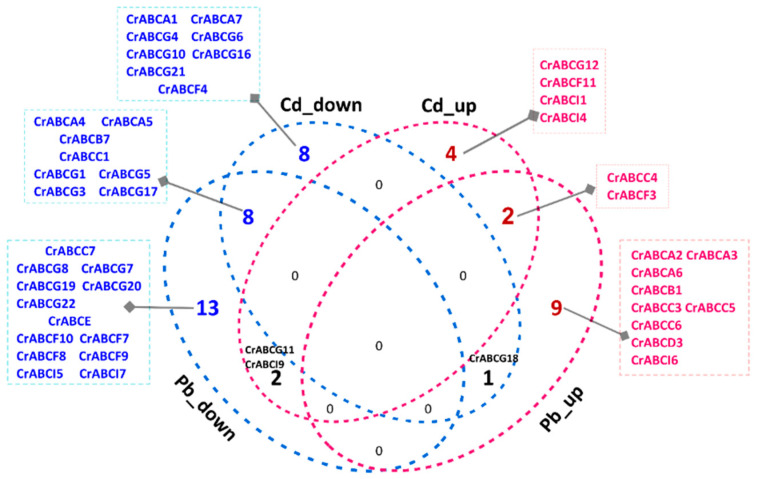
Comparison of differentially expressed *CrABCs* in response to Pb and Cd stress. Pb_down and Cd_down mean down-regulated *CrABCs* in response to Pb and Cd separately. Pb_up and Cd_up mean up-regulated *CrABCs* in response to Pb and Cd separately.

Microalgae have gained attention as suitable candidates for bioremediation, especially for the bio-concentration of heavy metals. As key members of the detoxification mechanism of heavy metal tolerance, ABCs play roles in the uptake, detoxification, and sequestration of heavy metals [77]. In this study, we found no differently expressed *CrABCs* under low-Pb stress (3 μM Pb treatment). On the contrary, 10 up-regulated and 23 down-regulated *CrABCs* responded to the high-Pb stimulation (80 μM Pb treatment) (Figure 6 and Table S5). For Cd stimulation, 8 *CrABCs* were induced in contrast to the 16 *CrABCs* that were repressed under the 12 μM Cd treatment (Figure 6 and Table S6). However, the Cd-related *CrABCC10* was not up-regulated in our investigation because *CrABCC10* was investigated under the 12 μM Cd treatment in this study, which is incomparable with the previous report using a 100 μM Cd treatment [11]. This suggests that *CrABCC10* is a critical responder for the mechanism of tolerance to extreme doses of Cd. By comparing the DEGs under Pb and Cd treatment, we found that the responsive ABCs might be distinct under different treatments. However, *CrABCC4* and *CrABCF3* were common responders for Pb and Cd (Figure 6). CrABCC4 was most homologous to AtABCC1 and AtABCC2 (Figure 2a), the known transporters for heavy metal uptake and sequestration in *Arabidopsis* [78]. Therefore, we speculated that *CrABCC4* is the most promising gene working in heavy metal resistance that requires further investigation.

#### 2.5.4. Nitrogen and Sulfur Starvation

Nitrogen (N) starvation and sulfur (S) starvation are the universal strategies that remodel carbon partitioning intracellularly to trigger the lipid accumulation of microalgae [79,80,81]. N- and S-deprived conditions lead to “nutrient sparing” of *C. reinhardtii* and induce specific genes following metabolic reprogramming [81,82]. Except for particular transporters for N or S acquisition, other induced transporters are pivotal for global metabolic reprogramming [82]. For example, nitrogen-starvation-induced *CrABCA4* transports substrates into the endoplasmic reticulum for lipid synthesis [12]. Here, we investigated the expression pattern of *CrABCs* throughout the course of either N or S depletion up to 48 h after starvation, when lipid accumulation is pronounced (Tables S7 and S8).

The expression patterns of *CrABCs* under nitrogen and sulfur starvation were quite distinct according to the hierarchical clustering results (Figure S5). However, the up-regulated patterns of four *CrABCAs*, i.e., *CrABCA2*, *CrABCA4*, *CrABCA5*, and *CrABCA7*, were conformable from 6 h to 48 h under N or S deprivation (Table 2). As a confirmed CrABC for lipid accumulation, *CrABCA4* was obviously induced at 6 h, 8 h, 24 h, and 48 h after both N and S deprivation. We found that *CrABCA5* showed a similar expression trend to *CrABCA4* (Pearson correlation index = 0.99) under either N or S depletion. The mRNA abundance of *CrABCA5* was almost equivalent to that of *CrABCA4* under N depletion [12]. Moreover, CrABCA5 and CrABCA7 were in the same sub-branch of AtABC10 (Figure 2a), which is the transporter for TAG overaccumulation in *Arabidopsis* seeds [29]. The similar expression trends and levels of *CrABCAs* may indicate that more CrABCAs potentially participate in lipid accumulation beyond *CrABCA4*. 

Except for ABCAs, we found that *CrABCB5*, *CrABCB6*, *CrABCB7*, *CrABCC4*, *CrABCC8*, *CrABCG2*, *CrABCG7*, *CrABCG9*, *CrABCG11*, *CrABCG15*, *CrABCG17*, *CrABCG21*, and *CrABCG26* shared similar up-regulated patterns under both N and S deprivation (Table 2). Furthermore, we noticed that CrABCG3 was a unique transporter that showed opposite expression patterns from 6 h to 48 h after N and S deprivation. Although we know the limits about their functions, the high expression of these *CrABCs* indicated their potential roles for metabolic remodeling under N or S starvation.

### 2.6. Localization Assay of Half-Size CrABCGs

**Figure 7:**
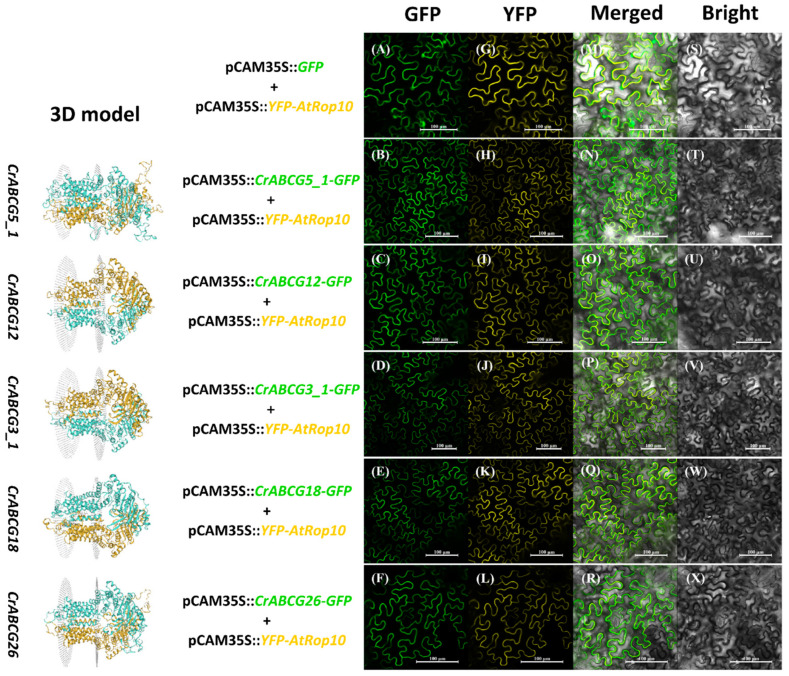
Subcellular localization of five half-size CrABCGs. pCAM35S::*YFP-AtRop10* is the positive marker showing yellow fluorescent signals to indicate the actual position of the plasma membrane in the tobacco leaf. pCAM35S::*CrABC-GFP* show green fluorescent signals to tell the exact location of each CrABCs in the tobacco leaf. (**A**–**F**), the green fluorescent signals of GFP or CrABC-GFP. (**G–L**), the yellow fluorescent signals of YFP-AtRop10. (**M**–**R**), the merged image of green fluorescent signals and yellow fluorescent signals of each assay. (**S**–**X**), the bright field of each assay. Length bar: 100 μm.

In higher plants, half-size CrABCGs localized in the plasma membrane participate in the secretion of lipidic compounds [52]. In this study, we cloned five half-size *CrABCGs*, namely *CrABCG5*, *CrABCG12*, *CrABCG3*, *CrABCG18*, and *CrABCG26*. Additionally, we analyzed the subcellular localization of these ABCGs using *Nicotiana benthamiana* leaves. These five half-size *CrABCGs* were fused in frame to the 5′-terminus of the *GFP* gene under the control of the CaMV (Capsicum Mottle Virus) 35S promoter (Figure S6). Co-expressed pCAM35S::*CrABCG-GFP* or pCAM35S::*GFP*, together with a YFP-labeled plasma membrane marker protein (pCAM35S::*YFP-AtRop10*) [83], were introduced into the epidermal cells of tobacco leaves. The pCAM35S::*GFP* signals were distributed in the cytosol, nucleus, and plasma membrane in epidermal cells, while the signals from five pCAM35S::*CrABCG-GFP*, merged with *YFP-AtRop10*, were concentrated in the plasma membrane of each assay (Figure 7). These results show that CrABCG5, CrABCG12, CrABCG3, CrABCG18, and CrABCG26 were half-size transporters localized in the cell membrane. *CrABCG3* and *CrABCG26* had significant responses to N or S starvation (Table 2). We speculated that they were putative transporters for lipid secretion.

## 3. Materials and Methods

### 3.1. Identification of ABC Genes

To identify ABC proteins of *C. reinhardtii*, we collected 45 CrABC proteins in PhycoCosm [17] and reannotated these proteins to Pfam with HMMER [84]. The information of 45 CrABC proteins is shown in Table S1. Finally, 44 CrABC proteins and all *Arabidopsis* ABC proteins [2] were used as queries to blast the proteome of the *C. reinhardtii* Genome v5.6 (JGI Project ID: 1084054) with a threshold e-value of 10^−5^. To confirm the conservative domain of putative ABC proteins, the putative proteins were annotated to the Pfam Database and Conserved Domain Database with default parameters in NCBI [85]. 

### 3.2. Sequence Analysis of ABC Genes

The gene information of putative *CrABCs* was obtained according to the protein ID of the Genome v5.6 using TBtools [86]. The exon–intron structures of *CrABCs* were mapped using the TBtools software [86]. The physical map of *CrABC* genes was drawn using Mapgene2chrom [87]. The subcellular localization of CrABC proteins was predicted using Plant-mPLoc [88]. The signal peptide and its cleavage sites of CrABCs were predicted using SignalP 5.0 [89]. 

### 3.3. Phylogenetic Analysis and Structure Analysis of ABC Proteins

Phylogenetic analysis was conducted to classify ABC proteins of *C. reinhardtii*. First, multiple alignments of ABC proteins were proceeded using the ClustalW program [90]. The phylogenetic analysis was performed by MEGA7.0 with following parameters: neighbor-joining tree method, pairwise deletion, and bootstrap with 1000 replicates [91]. TMD and NBD distributions of CrABCs were searched using HMMER [84]. The annotation and visualization of the trees and domain distribution were implemented in iTOL v6.0 [92].

### 3.4. Duplications and Selective Pressure Analysis of Paralogous Genes

MCScanX was used to analyze the duplication events of the *C. reinhardtii* genes with the following blast parameters: e-value of 10^−5^ and Blasthits of 10. The genome dot plot was visualized using TBtools [86]. The codons alignment of paralogous genes proceeded using MEGA7.0 [91]. The non-synonymous substitution rate (Ka), the synonymous substitution rate (Ks), and the Ka/Ks of paralogous pairs were calculated using DnaSP [93]. Sliding window analysis of the Ka/Ks ratios was carried out with a window size of 60 angstroms using SWAKK [94].

### 3.5. Transcriptome Data Processing

The RNA-seq data were downloaded from the NCBI SRA database using SRA Toolkit (https://github.com/ncbi/sra-tools, accessed on 26 December 2021). The sequencing quality was assessed using fastp [95]. After filtering the adaptor and low-quality reads, the remaining clean reads were mapped to Genome v5.6 (JGI Project ID: 1084054) with STAR [96]. FPKM (fragments per kilobase of transcript per million fragments mapped) was calculated using RSEM [97]. Differentially expressed genes (DEGs) required a fold change (FC) of >2 and an adjusted *p*-value of <0.05 using Benjamini–Hochberg correction in DESeq2 [98].

### 3.6. Subcellular Localization of CrABCGs

To analyze the subcellular localization of CrABCG5, CrABCG12, CrABCG3, CrABCG18, and CrABCG26, we designed specific primers to clone the full-length coding sequence of candidate *CrABCG*s (Table S9). The full-length CDS of *CrABCGs* was amplified from *C. reinhardtii* cDNA and then inserted into the GFP fusion expression vector (Figure S6) using a ClonExpress II One Step Cloning Kit (Vazyme, Nanjing, China). The location signals were analyzed in the leaf tissue of *Nicotiana benthamiana* after transfecting by *Agrobacterium tumefaciens* GV3101. Transient expression of the GFP fusion protein (CrABCG-GFP) was observed after about 48 h using a ZEISS LSM 710 NLO (Zeiss, Germany). The plasma membrane (PM) signals were visualized with YFP-labeled PM markers (YFP-AtRop10).

## 4. Conclusions

**Figure 8:**
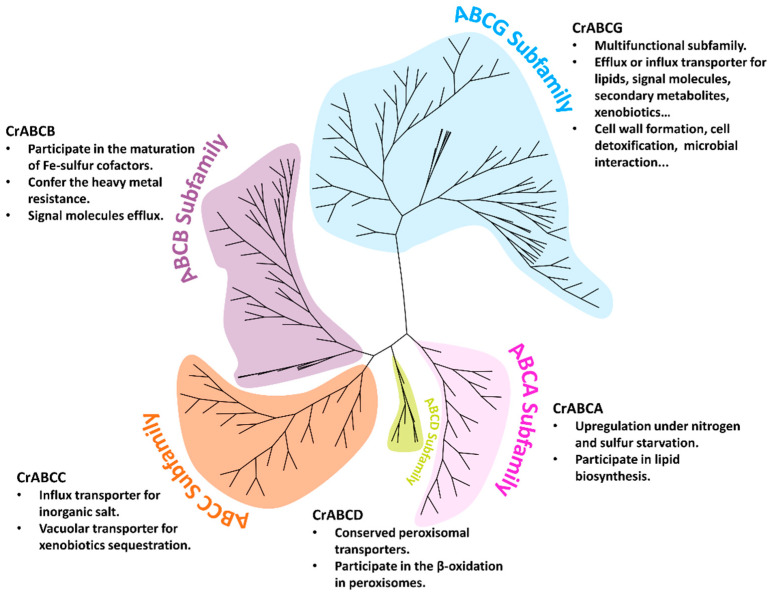
The putative functions of CrABC transporters. Subfamily ABCE, ABCF, and ABCI are not shown because they function as the accessory subunit in multi-subunit complexes that may participate in translating and catalyzing, except for transport.

Recently, transporter engineering has shown great potential in improving microalgae performance [15,99,100,101]. This study presented a comprehensive analysis of ABCs in *C. reinhardtii* for further microalgal transporters study. The CrABC proteins were classified into eight representative subfamilies. *CrABCs* did not massively expand or experience loss after ancient endosymbiosis events. Nevertheless, gene duplication events that occurred in small-scale regions gave rise to a few loci of *CrABCs*. The diversiform and promising roles of CrABCs were discussed according to the conservatively evolutionary relationships with AtABCs and their expression patterns in response to the adaptive environment. To sum up, we speculated the promising functions of microalgal ABCs by taking the subfamily as a unit (Figure 8). Moreover, five half-size CrABCGs were characterized as plasma membrane transporters, which might participate in lipid secretion in *C. reinhardtii*. Genome-wide identification of *C. reinhardtii* ABCs will allow us to gain a more comprehensive understanding of the functional evolution of this family. More profound knowledge of the transporter mechanism will be processed for the emerging and effective strategies to improve microalgae properties, including but not limited to metabolic improvement and heavy metal capture. The premise is that more microalgal transporters must be characterized in terms of localization, transport features, and specificity at the molecular level.

## Figures and Tables

**Table 1:** Subfamilies of ABC proteins in *C. reinhardtii* and other plants.

Subfamily	*Chlamydomonas* *reinhardtii*			Higher Plants ^c^			
	V3.0 ^a^	V5.6 ^b^	V5.6	Arabidopsis	Tomato	Rice	Barley
Total	69	44	75	130	154	134	131
ABCA	4	1	7	12	9	6	7
ABCB	8	6	8	30	29	27	32
ABCC	12	5	10	17	26	17	22
ABCD	1	3	3	2	2	2	4
ABCE	0	0	1	3	2	6	3
ABCF	0	8	11	5	6	6	5
ABCG	9	13	26	43	70	50	49
ABCI	7	8	9	16	10	16	9
Undefined	17	0	0	0	0	4	0

Note: ^a^, The subfamily data of Genome V3.0 were identified by Hwang et al. [2]. ^b^ There were 45 *CrABCs* published in PhycoCosm, 1 of which is a wrong annotation. ^c^ The number of transporters was obtained from previous studies: Arabidopsis and rice [5], tomato [18], and barley [19].

**Table 2:** Up-regulated *CrABCs* in response to nitrogen and sulfur starvation.

Subfamily	Systematic Name	Time Courses under Sulfur Starvation					Time Courses under Nitrogen Starvation				
		10 min	6 h	8 h	24 h	48 h	10 min	6 h	8 h	24 h	48 h
ABCA	CrABCA2	−0.59	**1.54**	**1.92**	**3.28**	**2.04**	−0.80	**1.01**	**1.19**	**1.80**	**1.67**
	CrABCA4	0.67	**1.46**	**1.66**	**3.90**	**2.49**	**1.56**	**1.08**	**1.24**	**3.37**	**3.01**
	CrABCA5	0.30	0.68	0.98	**2.44**	**1.38**	**1.45**	0.68	0.86	**3.04**	**2.48**
	CrABCA7	0.02	0.61	0.72	**2.00**	**1.31**	0.37	0.19	0.47	**1.41**	**1.30**
ABCB	CrABCB5	0.11	0.83	1.00	**2.53**	**1.84**	0.57	0.83	0.70	**1.24**	**1.40**
	CrABCB6	0.74	**1.51**	**2.37**	**2.40**	0.93	−0.26	0.30	−0.06	**2.13**	**2.03**
	CrABCB7	**1.18**	**1.29**	**1.70**	**3.98**	**3.33**	−0.41	**1.46**	**1.42**	**3.07**	**3.03**
ABCC	CrABCC4	−0.57	**1.44**	**1.48**	**2.49**	**2.93**	−0.88	**2.82**	**2.66**	**2.26**	**3.16**
	CrABCC8	0.31	0.56	0.73	**2.66**	**2.08**	0.81	0.38	0.55	**1.76**	**1.17**
ABCG	CrABCG2	0.89	**5.40**	**5.98**	**9.29**	**8.51**	0.52	0.34	0.60	**2.02**	**1.99**
	CrABCG3	**2.21**	**−2.32**	**−2.87**	**−5.06**	**−3.59**	**2.06**	**3.26**	**3.45**	**2.90**	**2.49**
	CrABCG7	**1.34**	0.65	0.78	**1.70**	**1.54**	0.20	0.52	0.09	**1.28**	**1.14**
	CrABCG9	0.01	0.52	0.71	**1.97**	**1.36**	0.56	**1.13**	**1.23**	**1.39**	**1.55**
	CrABCG11	0.91	**1.41**	**2.03**	**4.38**	**4.16**	**1.53**	**1.02**	**1.40**	**3.02**	**3.50**
	CrABCG15	−0.30	**1.00**	**1.15**	0.96	0.93	−0.66	**1.64**	**1.65**	**1.51**	**1.64**
	CrABCG17	**2.04**	**1.63**	**1.55**	**2.24**	0.96	**2.11**	**1.46**	**1.77**	**3.03**	**2.80**
	CrABCG21	**1.25**	**1.99**	**2.19**	**4.05**	**3.36**	**1.78**	0.88	1.00	**2.33**	**1.98**
	CrABCG26	**1.64**	**1.86**	**2.92**	**4.62**	**3.44**	**1.33**	−0.29	−0.16	**1.25**	**1.16**

Note: The data show the log_2_(fold change) value of each point versus the expression level at 0 h. DEGs were selected according to the thresholds of the absolute value of log_2_(fold change) ≥ 1, *p*-adjusted < 0.05. The significant down-regulated and up-regulated data are bold fonts labeled with blue and red.

## Data Availability

Data available within the article and its supplementary materials.

## Supplementary Materials

The following supporting information can be downloaded at: https://www.mdpi.com/article/10.3390/md20100603/s1, Figure S1. Genetic maps of ABC protein loci in the *C. reinhardtii* genome; Figure S2. The gene structures of *CrABCs*; Figure S3. Phylogenetic tree of ABCEs, ABCFs, and ABCIs; Figure S4. Genome dot plot of homologous *C. reinhardtii* genes; Figure S5. Overview of *CrABCs’* expression under nitrogen and sulfur starvation; Figure S6. Construction of the binary vector for subcellular localization assay; Table S1. Detailed information of ATP-binding cassette genes in *C. reinhardtii*; Table S2. The 128 *Arabidopsis* ABCs used in this study; Table S3. Expression pattern of *CrABCs’* underlying cell growth and development under daily rhythms; Table S4. Expression pattern of *CrABCs* under high-concentration CO_2_ conditions; Table S5. Expression pattern of *CrABCs* under lead stress; Table S6. Expression pattern of *CrABCs* under cadmium stress; Table S7. Expression pattern of *CrABCs* under nitrogen starvation; Table S8. Expression pattern of *CrABCs* under sulfur starvation; Table S9. List of primer sequences used in this study.
