# Supplementary material for: Identification and Characterization of ATP-Binding Cassette Transporters in Chlamydomonas reinhardtii
Source: Mar Drugs. 2022 Sep 25;20(10):603. doi: 10.3390/md20100603 (PMC9605142; doi:10.3390/md20100603)
Supplement: Supplementary file 1 [file marinedrugs-20-00603-s001.zip › Supplementary file 1.pdf]

Prediction of transmembrane helices in proteins

ABCA Subfamily

**Half-size Transporter**	**Full-size Transporter**
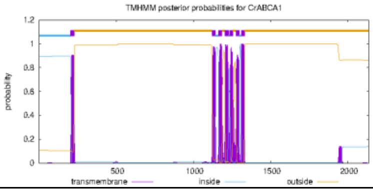	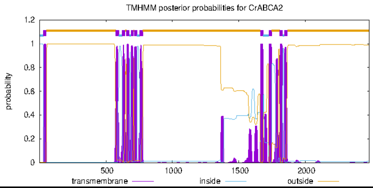
CrABCA1: Number of predicted transmembrane helices: 7	CrABCA2: Number of predicted transmembrane helices: 11
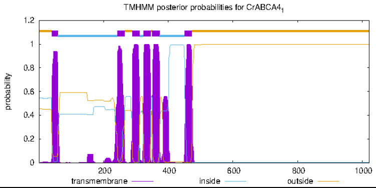	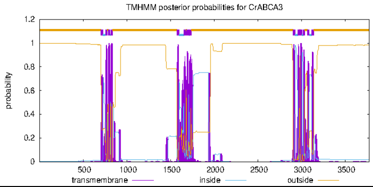
CrABCA4_1: Number of predicted transmembrane helices: 6	CrABCA3: Number of predicted transmembrane helices: 14
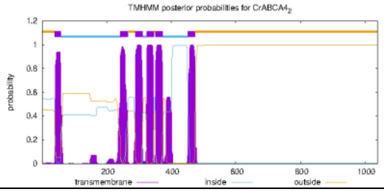	
CrABCA4_2: Number of predicted transmembrane helices: 6	
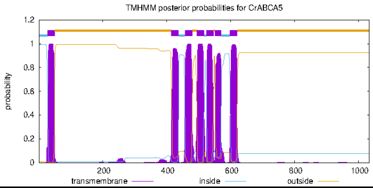	
CrABCA5: Number of predicted transmembrane helices: 7	
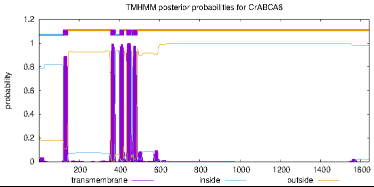	
CrABCA6: Number of predicted transmembrane helices: 5	
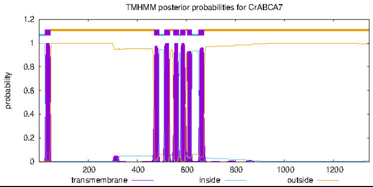	
CrABCA7: Number of predicted transmembrane helices: 7	

ABCB Subfamily

Half-size Transporter	Full-size Transporter
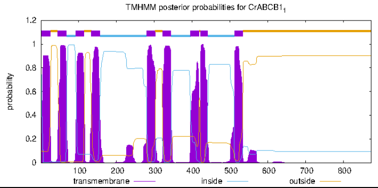	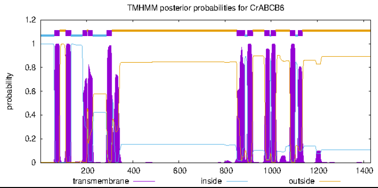
CrABCB1_1: Number of predicted transmembrane helices: 9	CrABCB6: Number of predicted transmembrane helices: 11
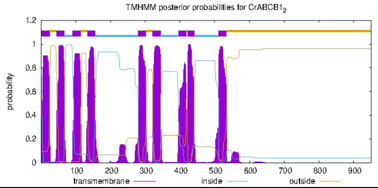	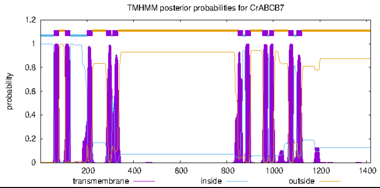
CrABCB1_2: Number of predicted transmembrane helices: 9	CrABCB7: Number of predicted transmembrane helices: 11
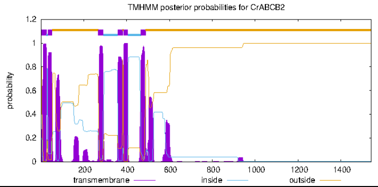	
CrABCB2: Number of predicted transmembrane helices: 6	
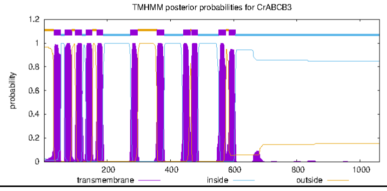	
CrABCB3: Number of predicted transmembrane helices: 11	
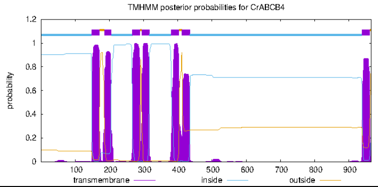	
CrABCB4: Number of predicted transmembrane helices: 7	
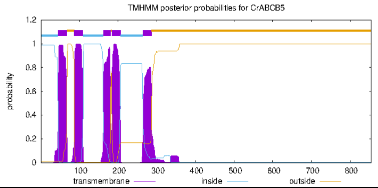	
CrABCB5: Number of predicted transmembrane helices: 5	
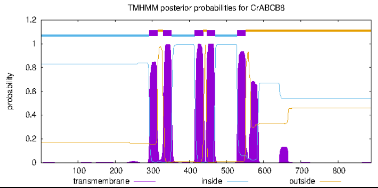	
CrABCB8: Number of predicted transmembrane helices: 5	

ABCC Subfamily

Half-size Transporter	Full-size Transporter
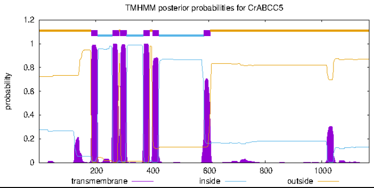	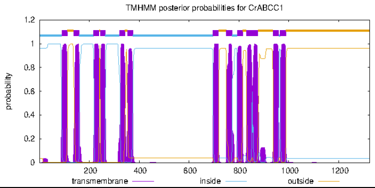
CrABCC5: Number of predicted transmembrane helices: 6	CrABCC1: Number of predicted transmembrane helices: 13
	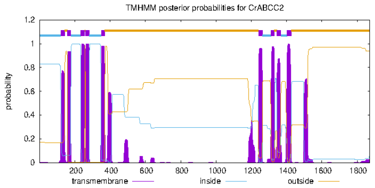
	CrABCC2: Number of predicted transmembrane helices: 9
	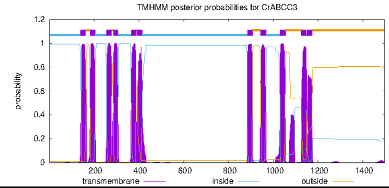
	CrABCC3: Number of predicted transmembrane helices: 11
	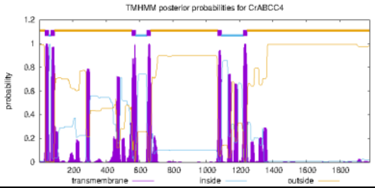
	CrABCC4: Number of predicted transmembrane helices: 6
	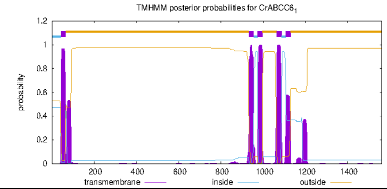
	CrABCC6_1: Number of predicted transmembrane helices: 5
	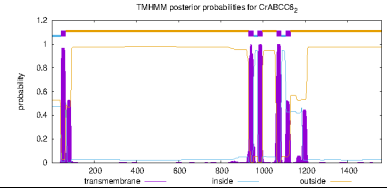
	CrABCC6_2: Number of predicted transmembrane helices: 5
	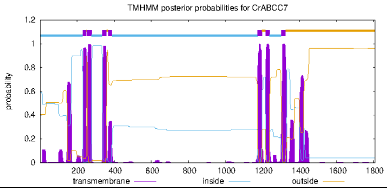
	CrABCC7: Number of predicted transmembrane helices: 7

	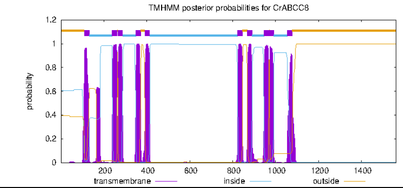
	CrABCC8: Number of predicted transmembrane helices: 10
	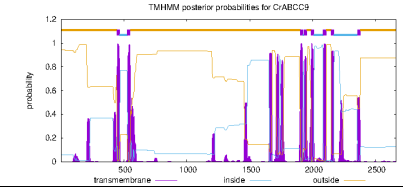
	CrABCC9: Number of predicted transmembrane helices: 8
	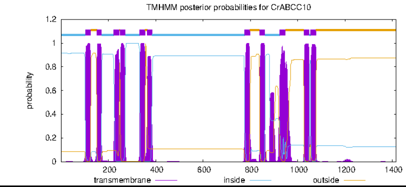
	CrABCC10: Number of predicted transmembrane helices: 11

ABCD Subfamily

Half-size Transporter	Full-size Transporter
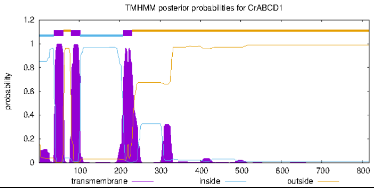	
CrABCD1: Number of predicted transmembrane helices: 3	
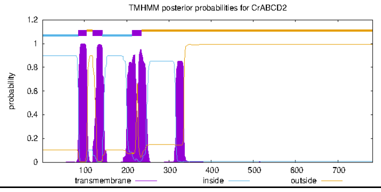	
CrABCD2: Number of predicted transmembrane helices: 3	
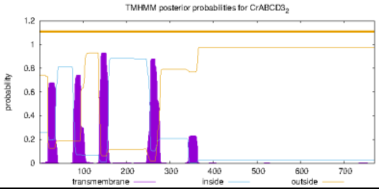	
CrABCD3_2: Number of predicted transmembrane helices: 0	
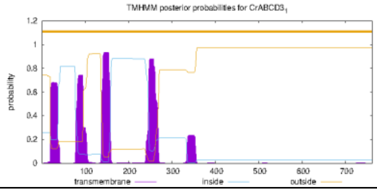	
CrABCD3_1: Number of predicted transmembrane helices: 0	
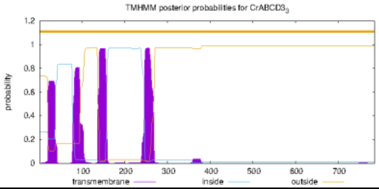	
CrABCD3_3: Number of predicted transmembrane helices: 0	

ABCE/F Subfamily

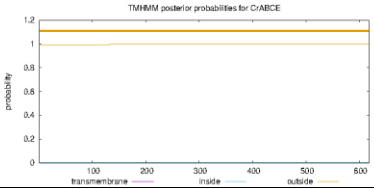	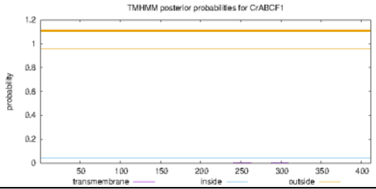
CrABCE: Number of predicted transmembrane helices: 0	CrABCF1: Number of predicted transmembrane helices: 0
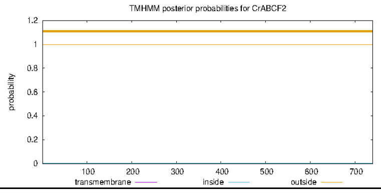	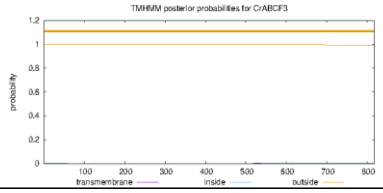
CrABCF2: Number of predicted transmembrane helices: 0	CrABCF3: Number of predicted transmembrane helices: 0

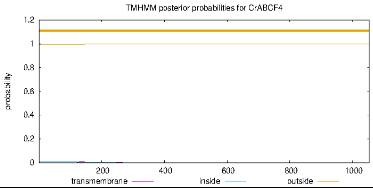	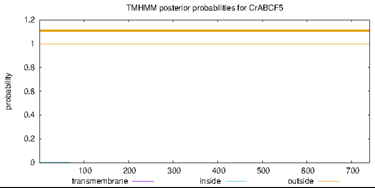
CrABCF4: Number of predicted transmembrane helices: 0	CrABCF5: Number of predicted transmembrane helices: 0
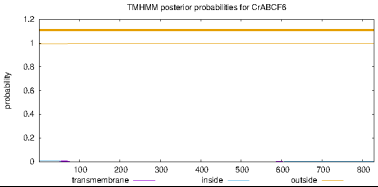	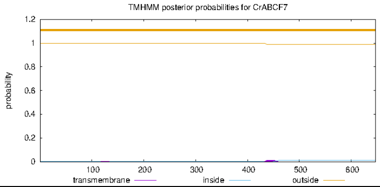
CrABCF6: Number of predicted transmembrane helices: 0	CrABCF7: Number of predicted transmembrane helices: 0
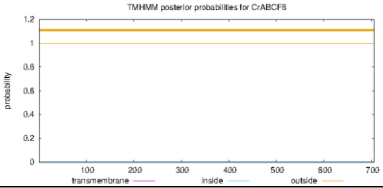	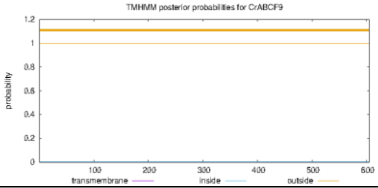
CrABCF8: Number of predicted transmembrane helices: 0	CrABCF9: Number of predicted transmembrane helices: 0
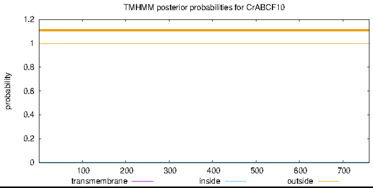	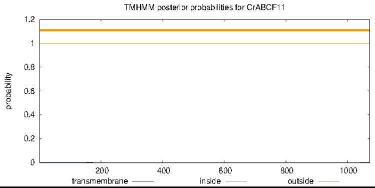
CrABCF10: Number of predicted transmembrane helices: 0	CrABCF11: Number of predicted transmembrane helices: 0

ABCG Subfamily

**Half-size Transporter**	**Full-size Transporter**
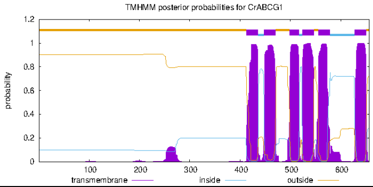	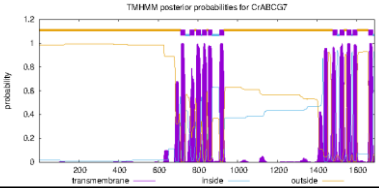
CrABCG1: Number of predicted transmembrane helices: 6	CrABCG7: Number of predicted transmembrane helices: 11
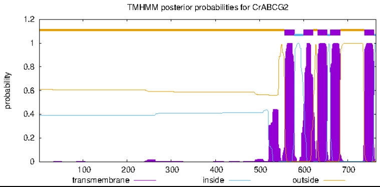	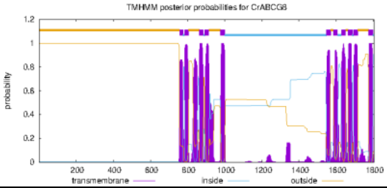
CrABCG2: Number of predicted transmembrane helices: 5	CrABCG8: Number of predicted transmembrane helices: 11
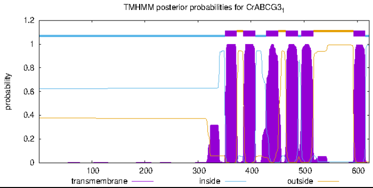	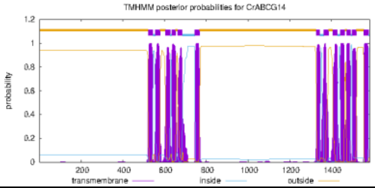
CrABCG3_1: Number of predicted transmembrane helices: 6	CrABCG14: Number of predicted transmembrane helices: 13

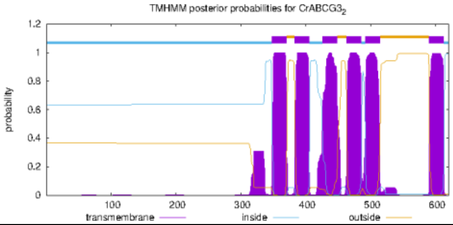

CrABCG3_2: Number of predicted transmembrane helices: 6

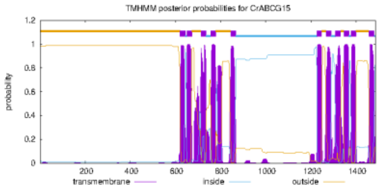

CrABCG15: Number of predicted transmembrane helices: 11

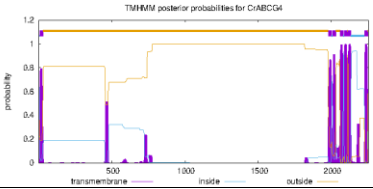

CrABCG4: Number of predicted transmembrane helices: 7

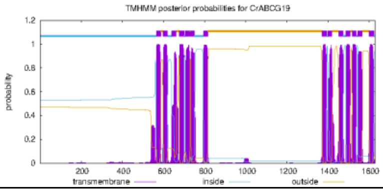

CrABCG19: Number of predicted transmembrane helices: 14

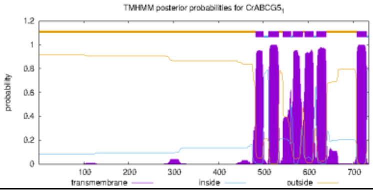

CrABCG5_1: Number of predicted transmembrane helices: 7

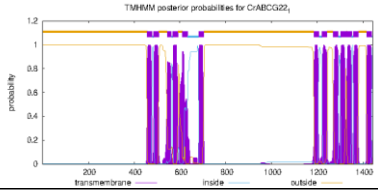

CrABCG22_1: Number of predicted transmembrane helices: 13

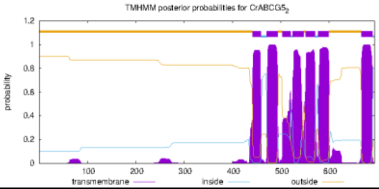

CrABCG5_2: Number of predicted transmembrane helices: 7

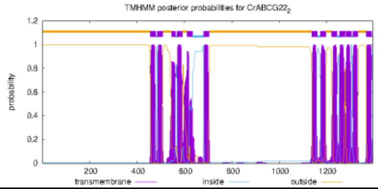

CrABCG22_2: Number of predicted transmembrane helices: 13

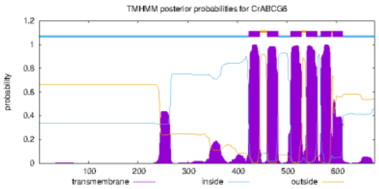

CrABCG6: Number of predicted transmembrane helices: 6

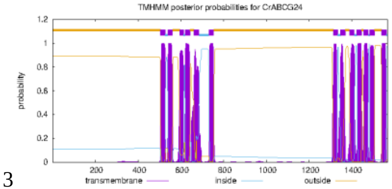

CrABCG24: Number of predicted transmembrane helices: 13

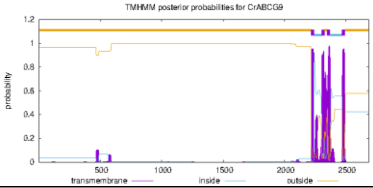

CrABCG9: Number of predicted transmembrane helices: 4

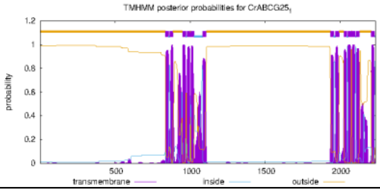

CrABCG25_1: Number of predicted transmembrane helices: 13

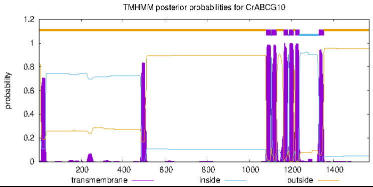

CrABCG10: Number of predicted transmembrane helices: 6

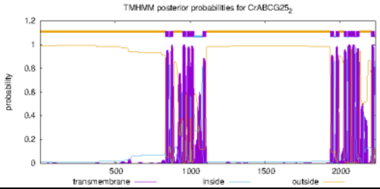

CrABCG25_2: Number of predicted transmembrane helices: 13

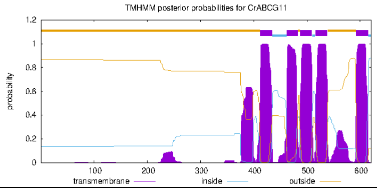

CrABCG11: Number of predicted transmembrane helices: 5

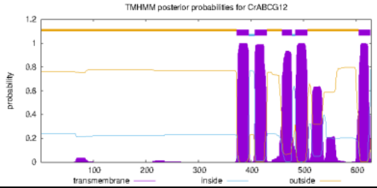

CrABCG12: Number of predicted transmembrane helices: 5

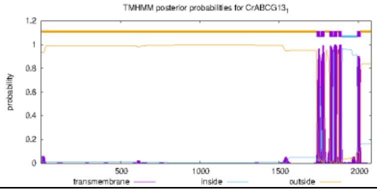

CrABCG13_1: Number of predicted transmembrane helices: 6

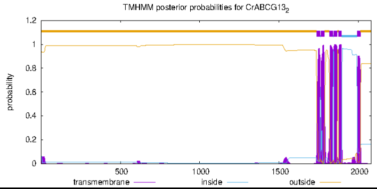

CrABCG13_2: Number of predicted transmembrane helices: 6

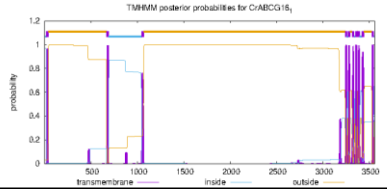

CrABCG16_1: Number of predicted transmembrane helices: 10

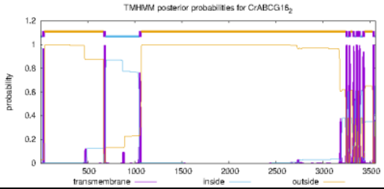

CrABCG16_2: Number of predicted transmembrane helices: 10

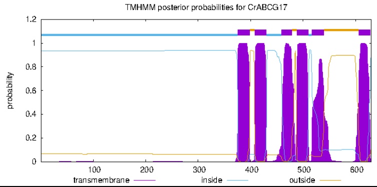

CrABCG17: Number of predicted transmembrane helices: 6

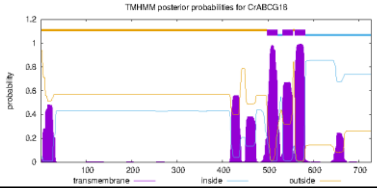

CrABCG18: Number of predicted transmembrane helices: 3

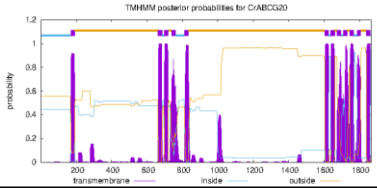

CrABCG20: Number of predicted transmembrane helices: 12

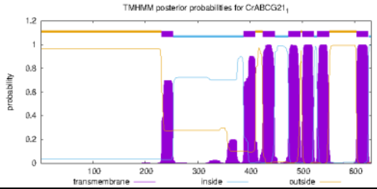

CrABCG21_1: Number of predicted transmembrane helices: 7

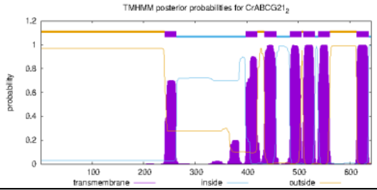

CrABCG21_2: Number of predicted transmembrane helices: 7

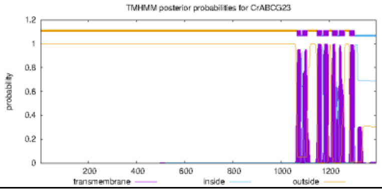

CrABCG23: Number of predicted transmembrane helices: 7

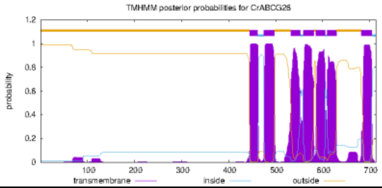

CrABCG26: Number of predicted transmembrane helices: 7

ABCI Subfamily

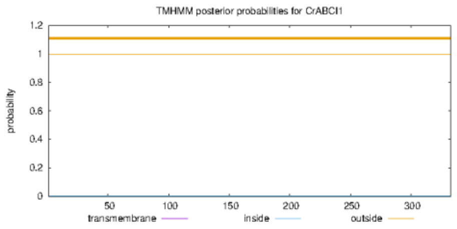

CrABCI1: Number of predicted transmembrane helices: 0

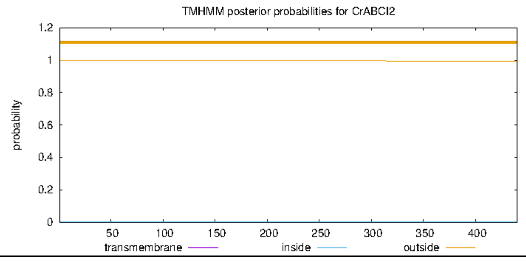

CrABCI2: Number of predicted transmembrane helices: 0

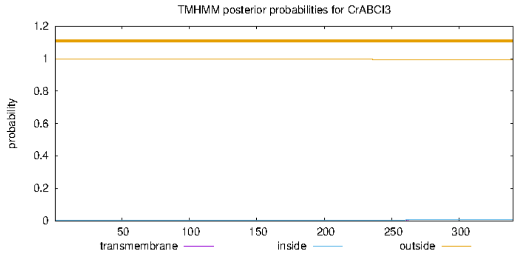

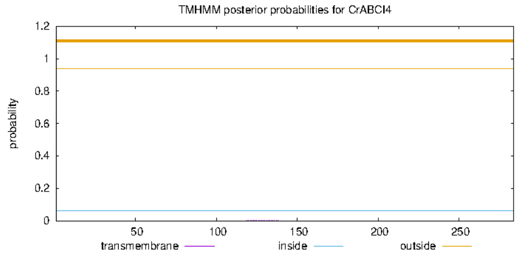

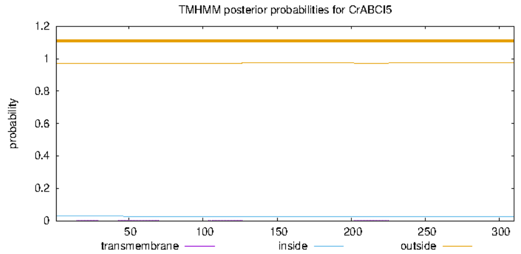

CrABCI3: Number of predicted transmembrane helices: 0

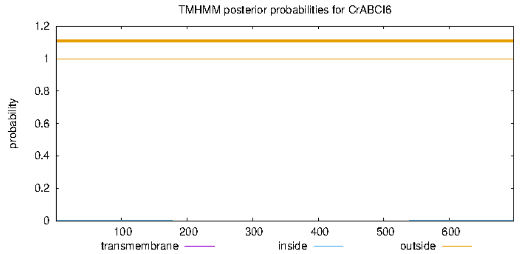

CrABCI4: Number of predicted transmembrane helices: 0

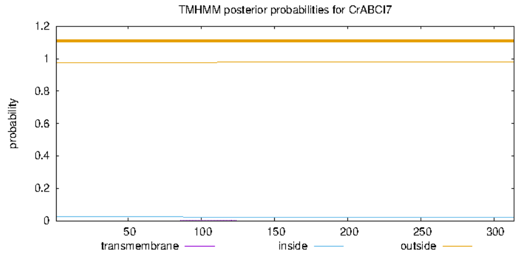

CrABCI5: Number of predicted transmembrane helices: 0

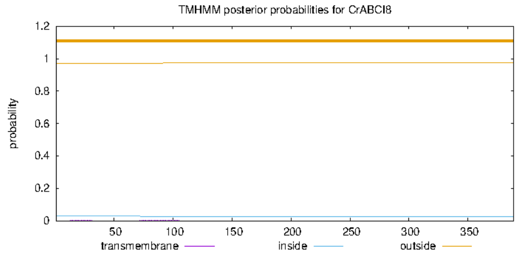

CrABCI6: Number of predicted transmembrane helices: 0

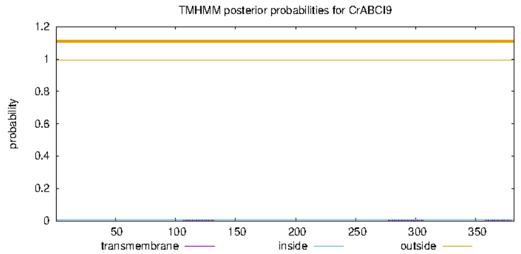

CrABCI7: Number of predicted transmembrane helices: 0

CrABCI8: Number of predicted transmembrane helices: 0

CrABCI9: Number of predicted transmembrane helices: 0
