# Supplementary figures and images for: Identification and Characterization of ATP-Binding Cassette Transporters in Chlamydomonas reinhardtii
Source: Mar Drugs. 2022 Sep 25;20(10):603. doi: 10.3390/md20100603 (PMC9605142; doi:10.3390/md20100603)

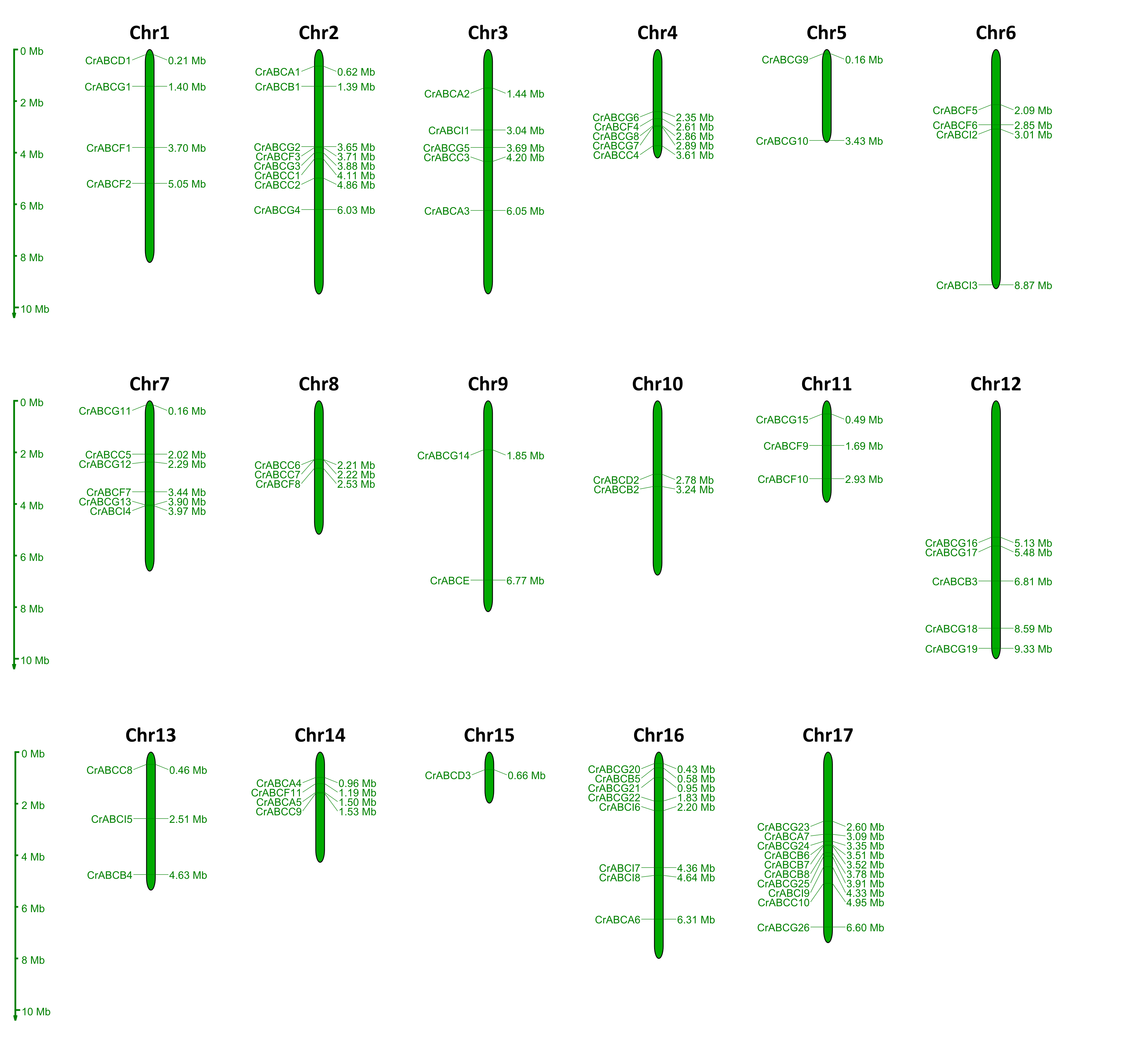

Supplement: Supplementary file 1 [file marinedrugs-20-00603-s001.zip › Figure S1.png]

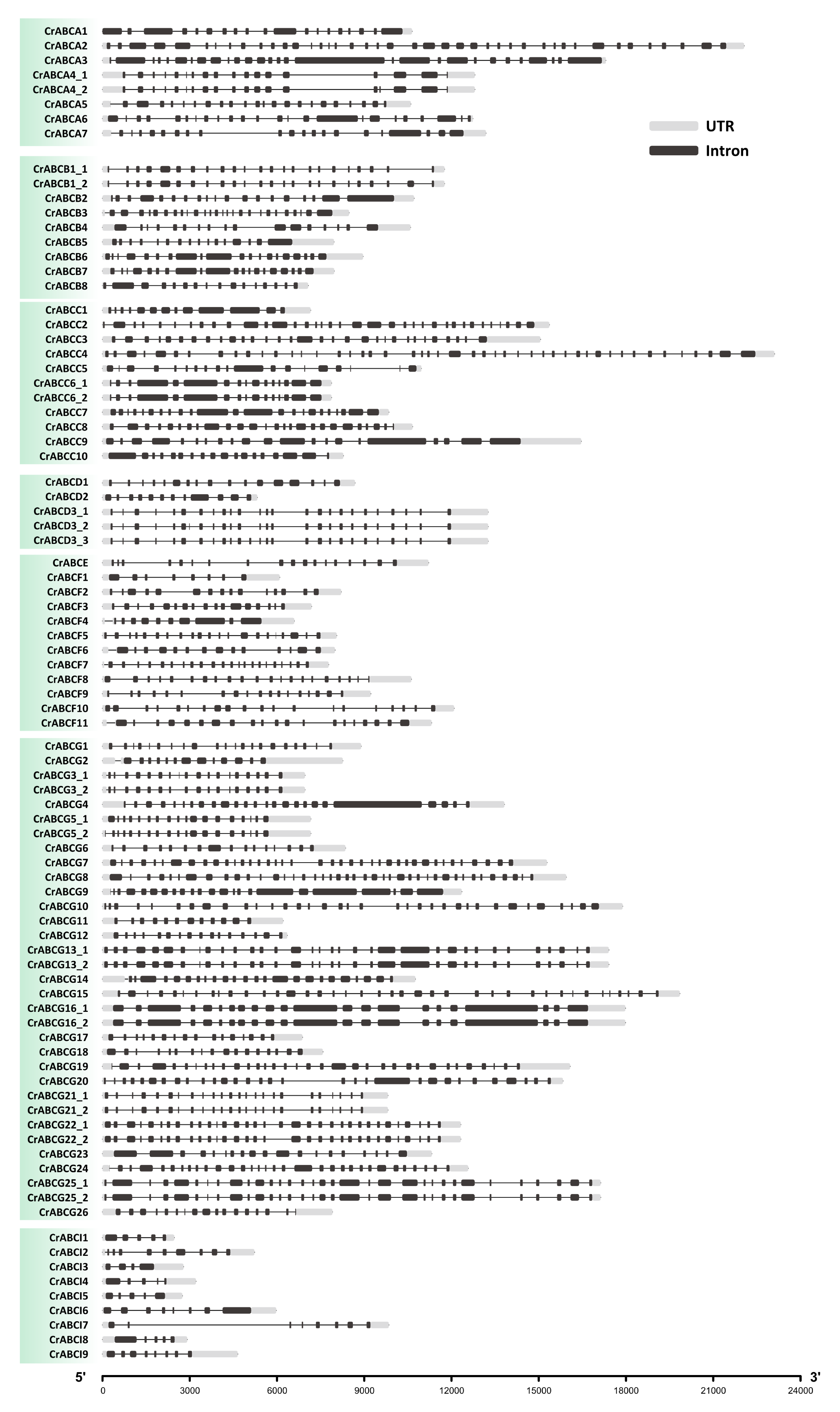

Supplement: Supplementary file 1 [file marinedrugs-20-00603-s001.zip › Figure S2.png]

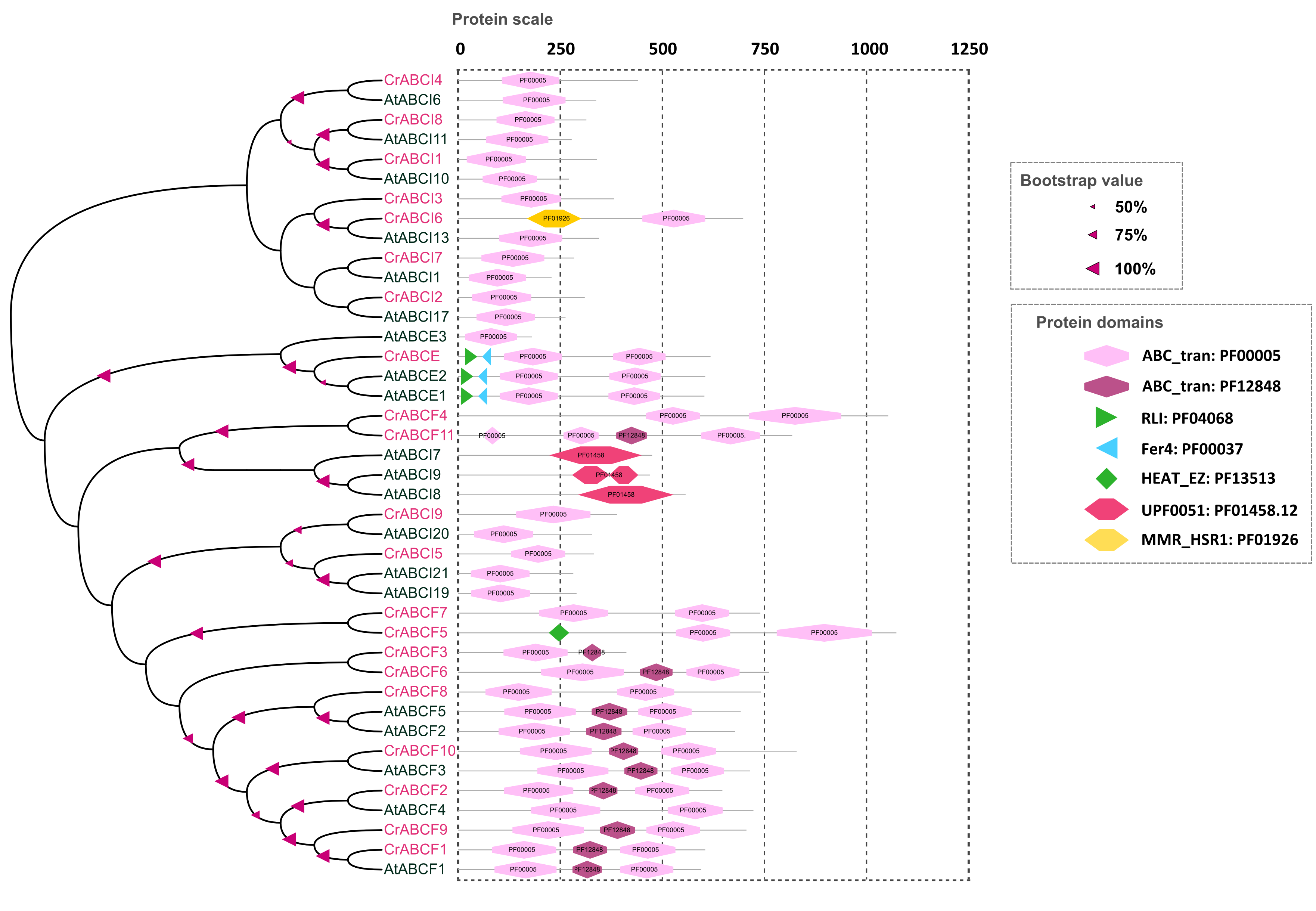

Supplement: Supplementary file 1 [file marinedrugs-20-00603-s001.zip › Figure S3.png]

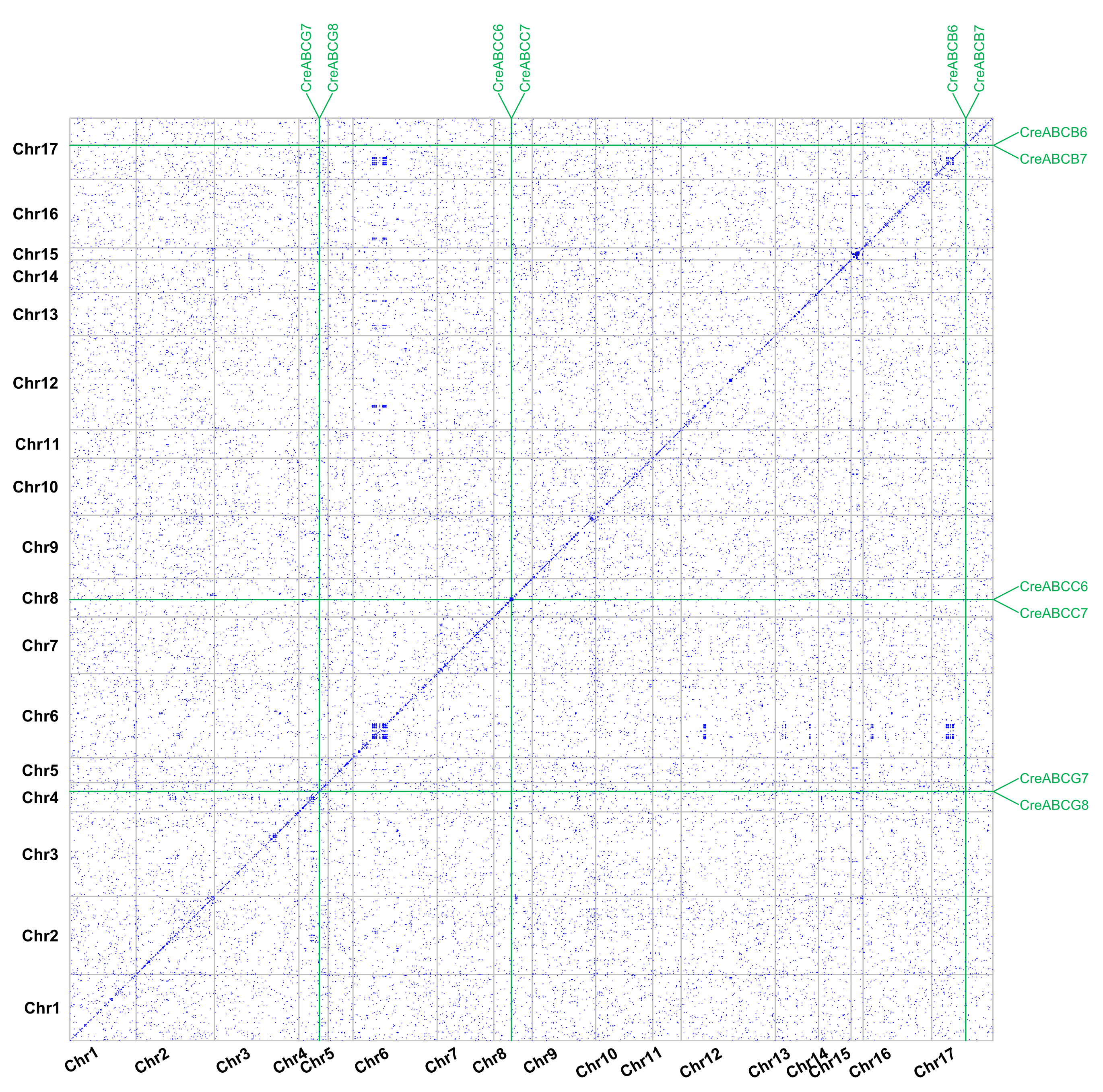

Supplement: Supplementary file 1 [file marinedrugs-20-00603-s001.zip › Figure S4.png]

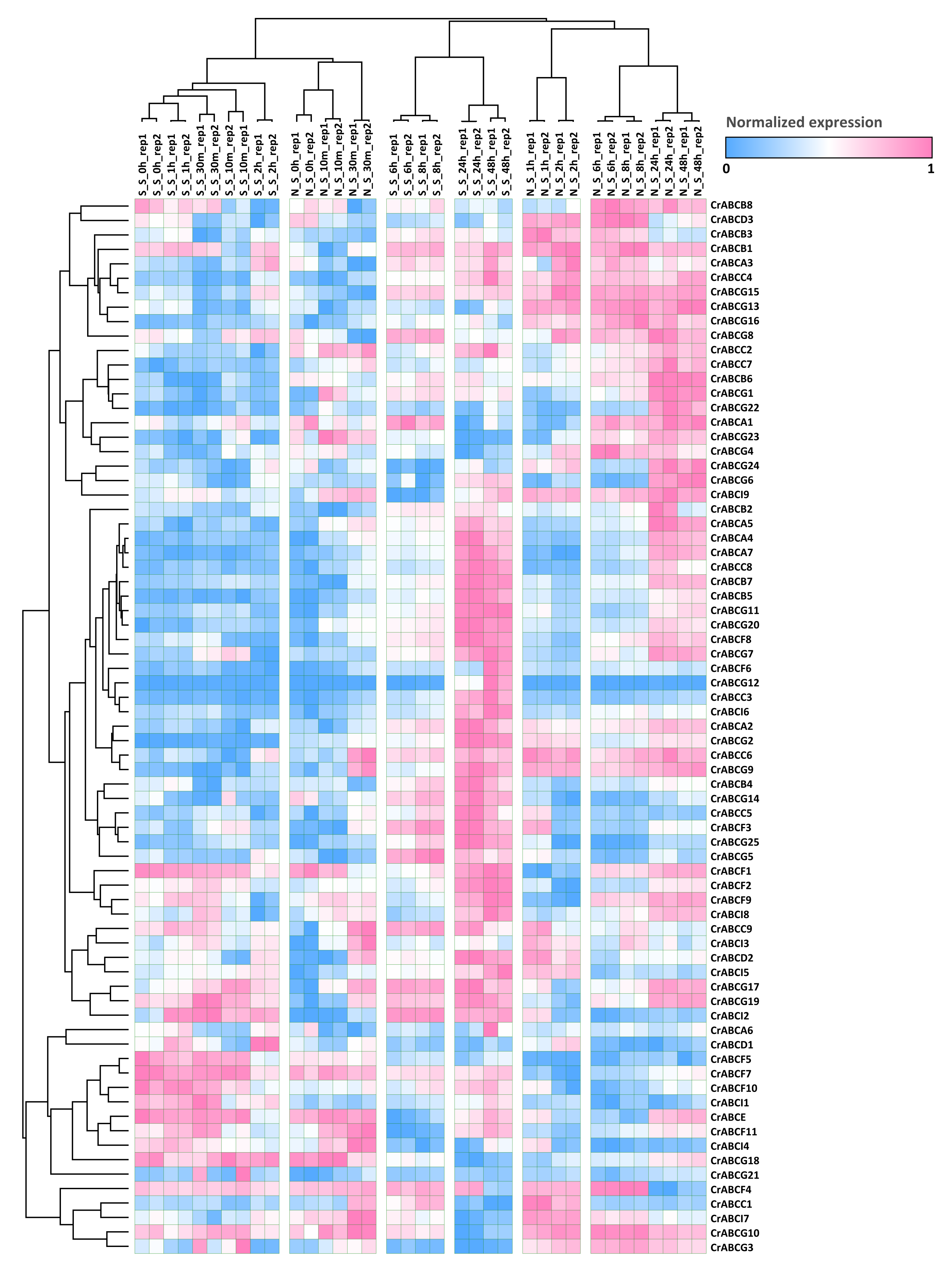

Supplement: Supplementary file 1 [file marinedrugs-20-00603-s001.zip › Figure S5.png]

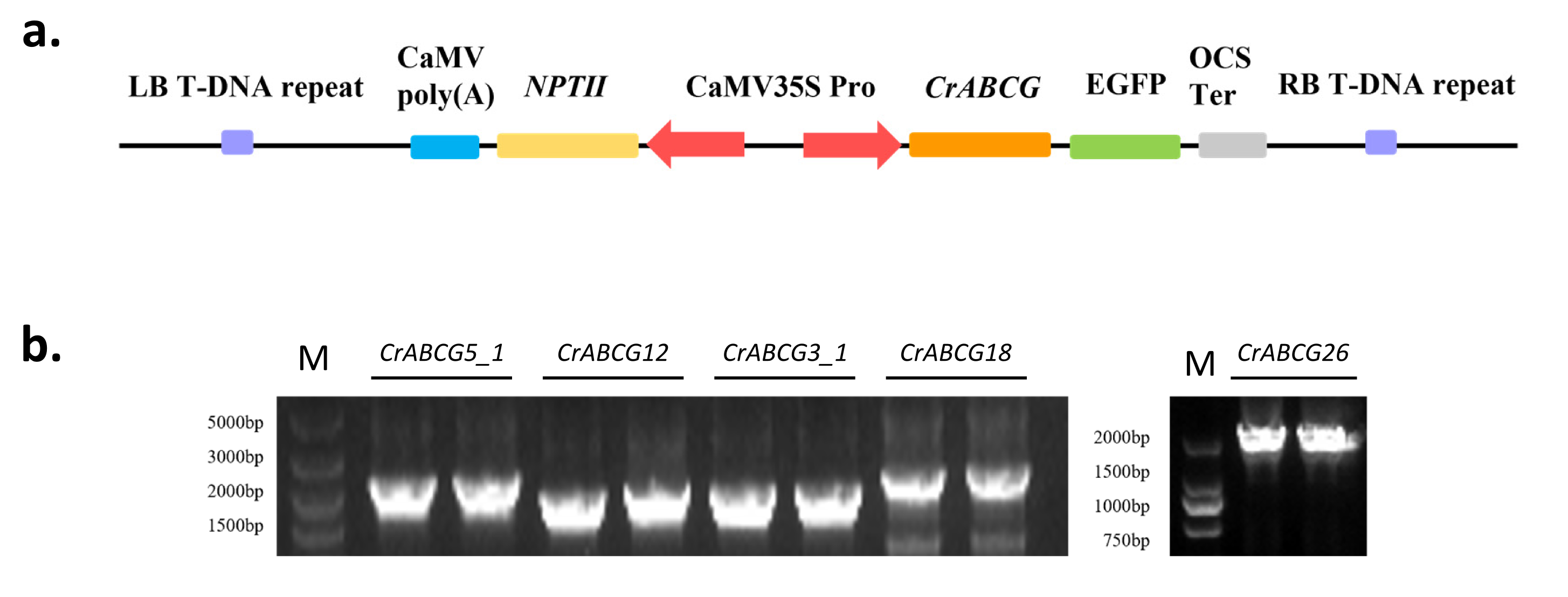

Supplement: Supplementary file 1 [file marinedrugs-20-00603-s001.zip › Figure S6.png]
